# PAS-Domain Protein Orientation at a Polyelectrolyte Surface Revealed by Infrared Nanospectroscopy, Chiral Vibrational Spectroscopy, and Molecular Dynamics Simulations

**DOI:** 10.1021/acs.biomac.5c02743

**Published:** 2026-04-30

**Authors:** Ferenc Bogár, Montserrat Román Quintero, János Horváth, Zoltán Násztor, Mark Mero, Szilvia Krekic, Alexander Veber, Ljiljana Puskar, András Dér, Zsuzsanna Heiner

**Affiliations:** † HUN-REN-SZTE Biomimetic Systems Research Group, Department of Medical Chemistry, 37442 University of Szeged, H-6720 Szeged, Hungary; ‡ School of Analytical Sciences Adlershof, 9373 Humboldt-Universität zu Berlin, 12489 Berlin, Germany; § Department of Chemistry, Humboldt-Universität zu Berlin, 12489 Berlin, Germany; ∥ HUN-REN Biological Research Centre, Institute of Biophysics, Institute of Biophysics, H-6726 Szeged, Hungary; ⊥ Doctoral School of Physics, University of Szeged, H-6720 Szeged, Hungary; # Max Born Institute for Nonlinear Optics and Short Pulse Spectroscopy, 12489 Berlin, Germany; ∇ Institute for Electronic Structure Dynamics, Helmholtz-Zentrum Berlin für Materialien und Energie GmbH, 12489 Berlin, Germany

## Abstract

PAS domains mediate protein–protein interactions that enable functions such as sensing, signaling, dimerization, and localization. The photoactive yellow protein (PYP) from *Halorhodospira halophila* is a model PAS-domain protein involved in negative phototaxis, yet its signaling partner remains unidentified. Here, we present a method to resolve protein orientations in PAS-domain signaling by combining nano-FTIR and chiral vibrational sum-frequency generation (VSFG) spectroscopy with molecular dynamics simulations and VSFG spectral calculations. As a demonstration, we used a charged homopolypeptide, poly-l-lysine (PLL), as a surrogate binding surface to probe PYP docking. We found that PYP adopts a preferred interfacial orientation driven primarily by dipole–dipole interactions, despite its water-soluble, i.e., cytoplasmic nature. Remarkably, the inferred interaction surface and orientation closely match those observed in PYP homodimers and in a CNBh-PAS heterodimer. This methodology enables in situ determination of protein orientational preferences during protein–protein interactions and may facilitate identification of binding partners in PAS-domain signaling pathways.

## Graphical abstract

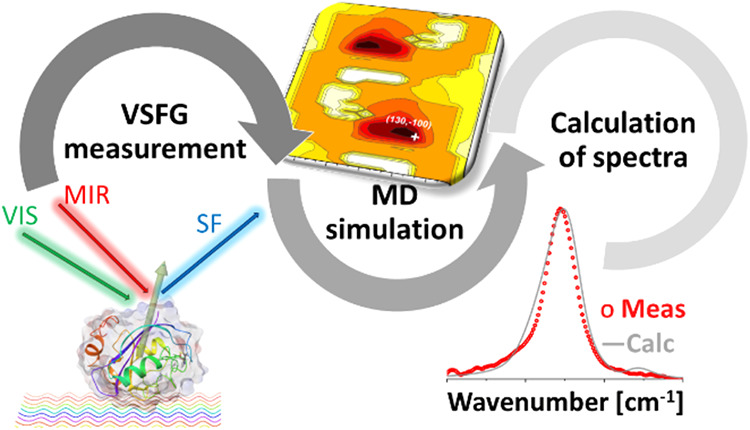

## 1 Introduction

Globular proteins often form functional complexes with other proteins via noncovalent protein–protein bonding. Each complex has a unique biological function that correlates with its quaternary structure. Such protein–protein interactions play a crucial role in many, if not most, biological processes in living organisms, from signal transduction through enzymatic and immune reactions to the assembly of cellular components.^1^ Recent studies have shown that abnormal protein–protein interactions can lead to neurodegenerative diseases, infections, and even cancer.^2,3^ Moreover, protein interactions with larger structures, such as membranes, supramolecular assemblies, and biocompatible surfaces, also have pivotal importance in signal transduction processes, folding-unfolding issues, pathophysiological mechanisms, and, e.g., determining the properties of the tissue-implant interface.^4^ Therefore, elucidating the elementary molecular mechanisms at the protein interface is vital for developing protein-based therapeutic agents, functional biomaterials, and biodiagnostic tools.

While protein–protein interactions are well characterized in bulk by X-ray crystallography and nuclear magnetic resonance, interfacial data remain scarce. Yet molecular structure and dynamics can differ markedly at interfaces compared to the bulk.^3,5^ X-ray and neutron-based techniques provide structural information only under highly controlled conditions, often unsuitable for strongly scattering, nonuniform protein layers. In such cases, symmetry- and vibrationally sensitive optical methods offer access to both secondary structure and orientation. Scattering-type scanning near-field optical microscopy (s-SNOM) combined with nano-FTIR spectroscopy enables infrared spectroscopic imaging with lateral resolution of a few tens of nanometers and depth sensitivity by detecting local optical near-field interactions between a metallic AFM tip and the sample.^6,7^ Nano-FTIR spectroscopy is primarily sensitive to out-of-plane vibrational dipole components through the vertical near-field polarization.^8^ Another optical technique, vibrational sum-frequency generation (VSFG) spectroscopy, selectively probes noncentrosymmetric, interfacial molecular vibrations with sensitivity to both molecular orientation and symmetry in situ and in a label-free manner.^9,10^ Its effective probing depth is set by the thickness of the noncentrosymmetric region, reaching down to a few angstroms, while its lateral resolution is limited by the wavelength of the spectrally narrowband upconversion beam employed in the technique. Even though the VSFG method was first employed in 1987,^11^ it has only recently been used for the characterization of molecular chirality^12–14^ and the secondary structure of proteins,^10,15,16^ which are crucial properties for a fundamental understanding of protein folding and protein interactions at interfaces. Polarization-resolved VSFG enables the determination of interfacial molecular orientation and chirality. The amide I region is widely used to probe protein secondary structure and surface alignment (ref 10), but its analysis is often complex.^17^ A common strategy is to compute VSFG spectra for all possible orientations and compare them with experiment; the best agreement identifies the most probable structure. This approach was applied by Boughton et al.^18^ to determine the orientation of α-helices at a lipid bilayer using polarization-dependent signal ratios.

In 2013, the excitonic amide I Hamiltonian model that had previously been developed for the calculation of IR and Raman spectra (for its detailed description, see ref 19) was adapted for calculating VSFG spectra, which facilitated these calculations even for large proteins.^20^ This model includes the coupling of amide I vibrations and the influence of H-bonds related to the amide groups.^20,21^ It can also be extended to include the spectral effects of structural fluctuations on the pico- and nanosecond time scales using molecular dynamics (MD) simulations for conformational sampling.^22^ Recently, Guo et al. developed a method^15,23,24^ that combines these two approaches with a new scoring method derived from the peak centers and widths of achiral VSFG spectra. This strategy was applied to determine the surface orientation of super uranyl binding protein,^23^ as well as GB1 protein and its mutants.^24^

In this paper, we address in situ protein–protein interactions by studying two proteinaceous partners paradigmatic of their type via combining experimental chiral VSFG spectroscopy and nano-FTIR spectroscopy with MD simulations and spectral calculations. The two partners are photoactive yellow protein (PYP) from *H. halophila*, a sensory protein containing the common “Per-Arnt-Sim (PAS) domain” ^25–27^ known for mediating protein–protein interactions,^28^ and a self-assembled thin film of poly-l-lysine (PLL), a naturally occurring polymer of the positively charged amino acid, lysine.^29^ The structure of PYP consists of a central β-sheet with five strands and helical connectors on both sides.^30,31^ The β-sheet sides contain two hydrophobic cores, which are expected to play a central role in protein–protein interactions. Homodimers and heterodimers based on PYP maintain the function of a PAS domain and, therefore, constitute a crucial model system.^32^ Nevertheless, the target protein for the signaling pathway originating from the photoexcited intermediate of PYP has so far not been identified.^33,34^ Furthermore, since PYP is a water-soluble protein, its orientation at the protein–protein interface is elusive. Here, the model partner of PYP is PLL, which is often used in bionanotechnological drug delivery due to its electrostatic interaction with negatively charged nucleic acids and proteins,^35^ and it is widely employed to cover stents and tissue-culture surfaces as a biocompatible cell-attachment coating.^36^ The well-known secondary structure of PLL helps unravel the favorable alignment of PYP at such an interface. As representative examples, we examine the structural similarities between our model system and two distinct protein architectures: the relatively compact PYP crystal structure and the substantially larger protein assembly, the KCNH voltage-gated K^+^ channel from mice.

## 2 Materials and Methods

### 2.1 Experimental Section

#### 2.1.1 Sample Preparation

CaF_2_ surfaces were functionalized using the spray-assisted layer-by-layer (LbL) method.^37^ First, nm-thick branched polyethylenimine (PEI, Sigma-Aldrich, molecular weight of 600,000–1,000,000) was deposited, which was followed by six pairs of poly-l-glutamic acid (PLGA, Sigma-Aldrich, molecular weight of 50,000–100,000 incl. sodium salt) and poly-l-lysine (PLL, Sigma-Aldrich, > 30,000 incl. hydrobromide). The concentration of the stock solutions was 5 mg/mL for PEI, and 1 mg/mL for PGA and PLL. After the deposition of PEI on the CaF_2_ surface, a few minutes of waiting time was applied, followed by a washing process with distilled water. In the next step, a PGA layer was deposited, and after a waiting period of a minute, the residue was thoroughly washed off. The buildup of this negatively charged PGA layer was followed by the deposition of a layer of PLL through another adding-waiting-washing cycle. Up to six pairs of PGA–PLL films were deposited on the PEI-functionalized CaF_2_ substrate. As is known from the literature, six pairs of layers are necessary for creating a very homogeneous surface.^38–41^ Based on our AFM studies (Figure S1), the thickness of the six-pair layer structure was ∼8 nm (Figure S2). The protein layer was created on the topmost PLL layer of the (PEI-(PLGA–PLL)_6_) assembled film by introducing 280 μM of PYP stock solution for a few minutes, followed by a washing period. This method resulted in a protein layer formation with a maximum thickness of 19 nm (Figure S2). After preparing the nm-thick films with and without the PYP layer, the so-coated CaF_2_ samples were placed in a homemade sample holder to provide a > 50% relative humidity environment and left to equilibrate for 10 min before the VSFG measurements. Use of the Si wafer results in low-roughness samples required for the nanoscale investigations, as well as ensures preferential sensitivity of the nano-FTIR technique to out-of-plane vibrational modes.^42^ For the s-SNOM measurements, the same protocol was used on a Si wafer because the high surface roughness of our CaF_2_ substrates prevented nanoscale investigations of the few-nm-thick samples. In the following, we refer to the PEI-(PLGA–PLL)_6_ structure as “PLL” and to the PEI-(PLGA–PLL)_6_+PYP structure as “PYP on PLL,” or simply “PYP.”

#### 2.1.2 Infrared Nanospectroscopy and Imaging

Infrared (IR) imaging and nanospectroscopy experiments were done at the IR-nanospectroscopy end-station at the IRIS beamline, BESSY II storage ring,^43^ using an s-SNOM (neaScope, Attocube GmbH, Germany) coupled to the broadband infrared synchrotron light.^44^ The measurements were performed in tapping mode with a Pt–Ir coated AFM-probe, with 25 nm of tip radius, ∼70 nm of tapping amplitude, and a resonance oscillation frequency of ≈ 266 kHz. The local nano-FTIR spectra were acquired by fixing the AFM tip at a specific sample location, and the spectra were collected in the range of 800–2100 cm^–1^ with a nominal spectral resolution of 8 cm^–1^. The typical acquisition time of a nano-FTIR spectrum was 12 min. For referencing the collected spectra, a spectrum from a clean part of the Si wafer was used.

The conversion of the recorded interferograms to infrared vibrational amplitude and phase spectra was performed by using an in-house script at the IRIS beamline with an asymmetric apodization window based on the three-term Blackmann-Harris function, and the measured signal was reference- and linear baseline-corrected to determine the optical phase spectra. The optical detector signal was demodulated at the second harmonic of the AFM cantilever oscillation frequency to suppress the far-field contribution, yielding optical amplitude and phase data used to reconstruct the images and spectra. Preprocessing of the simultaneously collected s-SNOM images, i.e., topography and mechanical phase, was performed in Gwyddion.^45^ All measurements were performed at room temperature with continuous dry nitrogen purging of the s-SNOM instrument that is sitting inside an acoustic enclosure.

#### 2.1.3 Vibrational Sum-Frequency Generation Spectroscopy

The apparatus is a home-built broadband VSFG spectrometer described in detail elsewhere.^46–48^ The driving laser was a 6 W, 100 kHz Yb/KGd­(WO_4_)_2_ laser oscillator-amplifier system operating at 1028 nm. The pump pulses were split into two arms. One arm was used to produce 5 μJ narrowband (picosecond) visible (VIS) pulses at 514 nm or 0.5 μJ narrowband near-infrared (NIR) pulses at 1028 nm, while the second arm delivered 0.7 μJ and 0.2 μJ broadband mid-infrared (MIR) pulses in the 2750–3800 cm^–1^ and 1400–1700 cm^–1^ spectral ranges to cover the C–H, N–H, and O–H stretching region and the amide I and II regions, respectively. A home-built purge box maintaining a consistent dry airflow was used for the MIR beamline to minimize water vapor absorption. The VIS/NIR and MIR pulses were spatially and temporally overlapped on the sample, and the reflected SFG signal was collected by a spectrograph equipped with a Peltier-cooled CCD. The spectral resolutions were ∼3 cm^–1^ and ∼5 cm^–1^ for applying the VIS and NIR narrowband beams, respectively. The VSFG spectra were collected in PPP, SSP, and SPP polarization combinations (the order of polarizations corresponds to the SFG, VIS/NIR, and MIR beams, respectively) in the C–H/N-H/O–H stretching range, while the spectra in the amide I region were obtained in SSP and SPP polarization. Measurements for each polarization combination were performed on three different samples, with 10–20 sample positions per sample. The measurements were carried out at a room temperature of 23 °C.

#### 2.1.4 Experimental VSFG Data Processing

After frequency calibration,^49^ the difference spectra were calculated by subtracting the spectrum without MIR excitation, i.e., the background spectrum. Each difference spectrum was divided by the applied acquisition time, which allowed us to convert the spectral intensity of each spectrum into a count-per-second (cps) unit. The nonresonant (NR) spectrum was collected from a silver surface and normalized with the applied infrared intensity. This normalization method and the usage of the cps unit allowed us to quantitatively compare the vibrational amplitudes of various spectral ranges, i.e., for spectral stitching. Each difference spectrum was normalized with this corrected NR spectrum at a given polarization combination. The resulting calibrated VSFG spectra at each polarization combination, the so-called effective value of |χ^(2)^|,^2^ were averaged and used for further spectral analysis. In the next step, the imaginary part of the effective χ^(2)^ was calculated based on the maximum entropy method (MEM),^50,51^ which enabled the extraction of the relevant Lorentzian oscillator resonance frequencies together with the probable relative signs of the corresponding amplitudes. This information was, in turn, used for spectral fitting of the normalized VSFG spectra by the sum of Lorentzian functions

1 $$I_{\mathrm{VSFG}}\left(\omega \right)\propto {\left|A_{\mathrm{NR}}e^{i\Phi }+\sum_{i=1}^{\nu }\frac{B_{\upsilon }}{\omega -\omega_{\nu }-i\Gamma_{\upsilon }}\right|}^{2}$$

where the first term on the right accounts for the NR contribution with an amplitude *A*_NR_ and a phase Φ, making it possible to describe both constructive and destructive interference. *B_ν_*, ω*_ν_*, and Γ*_ν_* are the amplitude, frequency, and damping factor of the ν^th^ Lorentzian resonance, respectively. In the data analysis, thin film (Fabry–Perot) interference effects were not considered, since the optical thickness of our samples was negligible compared to any of the laser wavelengths in the VSFG experiment.^52^

### 2.2 Molecular Dynamics Simulations

#### 2.2.1 Calculation of VSFG Spectra

The theoretical chiral (SPP) VSFG spectra of the investigated protein in the amide I region were calculated using the method described by Roeters et al.^20^ This method is based on the local mode description of the molecular vibrations in which the collective modes of the system are expanded based on local amide I vibrational modes using separate local coordinates for each amide group of the main chain of the protein. The local amide I vibrations are coupled, forming delocalized vibrational excitons described by the following Hamiltonian

2 $$H=\sum_{i=1}^{N}\hbar \omega_{i}^{0}b_{i}^{+}b_{i}+\sum_{\begin{array}{l} i,\;j=1\\ i>j \end{array}}^{N}\kappa_{\mathrm{ij}}\left(b_{i}^{+}b_{j}+b_{j}^{+}b_{i}\right)$$

where *N* is the number of residues in the protein chain, *b*_*i*_^+^, *b*_*i*_, andω_*i*_^0^ are the creation and annihilation operators and the frequency belonging to the local mode *i*, respectively. Further, κ_*ij*_ is the coupling constant between local modes *i* and *j*. This approach assumes that the amide I modes are side-chain independent and are mainly determined by the secondary structure of the protein. The coupling constants of these oscillators are approximated as the interaction energy of coupled transition-dipole moments.^53^ However, this approximation does not work properly for the nearest neighbors (κ_*i*,*i* ± 1_, κ_*i* ± 1,*i*_) in a polypeptide chain, because these residues are mechanically coupled through the bonds. Therefore, the conformation dependent values of these coupling constants were directly determined from density functional full normal mode calculations by Ham et al. ^47^ using the Hessian matrix reconstruction method.^19,54,55^ rather than using the simplified dipole–dipole coupling model. For this calculation, a glycine dipeptide analog was used as the simplest peptidic model system.^47^ The ω_*i*_^0^ = Ω^0^ – *δω*_*Pro*_ – *δω*_*HB*,*i*_ frequencies are calculated from the isolated local mode frequency Ω^0^ using a red-shift for proline residues (*δω*_*Pro*_) and another shift of *δω*_*HB*,*i*_ that originates from the hydrogen bonds formed between the main-chain amide groups and the H-bond donors and acceptors of the protein and the solvent. *δω*_*HB*,*i*_ can be calculated using the empirical formula suggested by Hamm et al.^53^ and the parametrization published by Roeters et al.^20^ and Meister et al.^56^

From the eigenvalues (ω*^ν^*) and eigenvectors *|ν*⟩ of the excitonic Hamiltonian, the frequency-dependent hyperpolarizability tensor of the protein can be calculated as

3 $$\beta_{\mathrm{ijk}}^{\left(2\right)}\left(\omega_{\mathrm{IR}}\right)=-\frac{1}{2\hbar }\sum_{\nu }\frac{\mu_{k}^{\nu }\alpha_{\mathrm{ij}}^{\nu }}{\left(\omega^{\nu }-\omega_{\mathrm{IR}}-i\Gamma \right)},$$

where μ_*k*_^ν^ is the transition dipole vector and α_*ij*_^ν^ the Raman tensor calculated for the υ-th normal mode of the excitonic system. These quantities can be calculated from the transition dipole vector and transition polarizability tensor of local amide I modes using the eigenvectors of the excitonic Hamiltonian in eq 2 as described by Roeters et al.^20^ Γ is the line width of the Lorentzian, which is assumed to be equal for all modes. The elements of the second-order susceptibility tensor can be obtained from *β*_*ijk*_^(2)^ as

4 $$\chi_{\mathrm{IJK}}^{\left(2\right)}\left(\omega_{IR}\right)=N\sum_{\begin{array}{l} i,\;j,\;k\;=\\ x,\;y,\;z \end{array}}\left\langle \left(I,i\right)\left(J,j\right)\left(K,k\right)\beta_{\mathrm{ijk}}^{\left(2\right)}\left(\omega_{IR}\right)\right\rangle .$$

Here, *I*, *J*, and *K* denote the coordinates in the laboratory (X, Y, Z), and *i*, *j*, *k* in the molecular frame (x, y, z) (see Figure 1).

**Figure 1:**
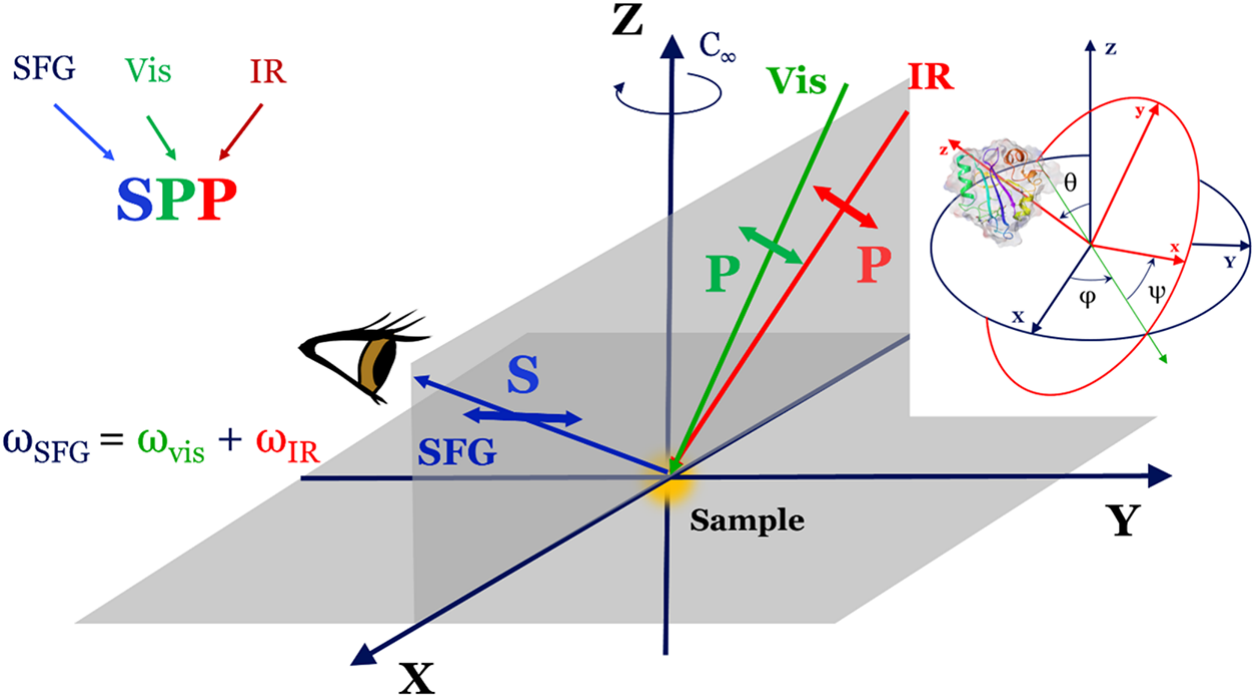
Chiral VSFG spectroscopy setup in SPP polarization combination and the definition of Euler angles that give the orientation of the protein-fixed coordinate frame (x, y, z) in the laboratory frame (X, Y, Z).

The same letters in bold are the unit vectors of the corresponding coordinate axes. The angle brackets ⟨⟩ indicate an average of the molecular geometries present in the sample, including the possible orientations on the surface and the large variety of intramolecular and intermolecular interactions. The effective second-order susceptibility tensor belonging to the SPP setup is

5 $$\chi_{SPP}^{\left(2\right)}\propto \cos\left(\rho_{vis}\right)\sin\left(\rho_{\mathrm{IR}}\right)L_{YY}\left(\omega_{sum}\right)L_{XX}\left(\omega_{vis}\right)L_{ZZ}\left(\omega_{\mathrm{IR}}\right)\chi_{YXZ}^{\left(2\right)}+\sin\left(\rho_{vis}\right)\cos\left(\rho_{\mathrm{IR}}\right)L_{YY}\left(\omega_{sum}\right)L_{ZZ}\left(\omega_{vis}\right)L_{XX}\left(\omega_{\mathrm{IR}}\right)\chi_{YZX}^{\left(2\right)}$$

where *L*_MM_ (M = X, Y, Z) denotes the Fresnel factors calculated for the sum-frequency, the visible, and the IR beams.^57^ After averaging for the rotation around the Z axis

6 $$\chi_{SPP}^{\left(2\right)}\propto \chi_{\mathrm{YZX}}^{\left(2\right)}-\chi_{\mathrm{XZY}}^{\left(2\right)}$$

From eq 6, the intensity of the SFG signal is

7 $$I\propto {\left|\chi_{\mathrm{SPP}}^{\left(2\right)}\right|}^{2}.$$

#### 2.2.2 Simulation Details and Sampling

An acceptable sampling of the conformational ensemble of the solvated protein and the protein–surface complex is inevitable to obtain a good-quality calculated spectrum. To this end, we used MD simulations with classical force fields. Namely, the OPLS4 force field^58^ was applied for the protein and surface parametrization, and the nonpolarizable SPC water model for the solvent, as implemented in the Desmond program of the Schrödinger package.^59,60^ The Nose-Hoover Chain thermostat and Martyna–Tobias–Klein barostat were applied to control the temperature and pressure, respectively.^61^ The RESPA integrator^62^ was used with a time step of 2 fs during the simulation. Long-range electrostatics were treated with the Particle Mesh Ewald (PME) method.^63^ A 200 ns NPT molecular dynamics simulation was performed, and evenly spaced samples were collected from the second half of the computation. Based on pioneering works by Roeters et al.^20,64^ and other closely related publications in this field,^19,56,23,15,24,65^ we have developed a Python code for the calculation of VSFG spectra using the MDAnalysis package^66,67^ to process MD trajectories. The 3D structure of PYP, obtained by high-resolution neutron crystallography, has been downloaded from the Protein Data Bank (PDB ID: 2ZOI).^68^

The orientation of the protein on the surface is characterized by the Euler angles φ, θ, and ψ (see Figure 1). We assume that the sample is optically isotropic in the XY plane; therefore, we average over the possible orientations in this plane, that is, for the φ angle. In this way, the different orientations of the protein can be distinguished by angles θ and ψ.

### 2.3 HADDOCK Docking

In order to estimate the binding quality of PYP at the top of the PLL–PGA layered film, a simplified protein complex refinement protocol, as implemented in the integrative modeling software package, HADDOCK, version 2.4, was used.^69–71^ There are three stages in the HADDOCK protocol by default. Initially, each protein is handled as a rigid entity during rigid-body docking (stage 1). Typically, experimental or anticipated interaction data serve as guidance for this docking stage. This data is converted into distance constraints that favor particular protein sections to bind without defining their relative orientation. The top models from stage 1 (200 by default) then advance to stage 2, where protein–protein interactions are further refined using a simulated annealing technique in torsion angle space. In order to maximize intermolecular interactions and permit minor conformational changes, flexibility is progressively added to the protein interface, initially for side chains and subsequently for both side chains and the backbone. The final stage (stage 3) refines the protein–protein interface by either an explicit solvent-shell molecular dynamics simulation or an energy minimization, which is the default in HADDOCK2.4. In our case, we used a simplified protocol that omits the randomization of the initial orientations of PYP and halves the number of different PLL–PYP complexes passed between stages.

## 3 Results and Discussion

### 3.1 PYP Adsorption at the Air-PLL Interface as Revealed by Chiral VSFG Spectroscopy

Figure 2A–I show the measured chiral (SPP) VSFG spectra of PLL and PYP adsorbed on the PLL surface in the C–H/O-H/N–H stretching and the amide I region. Figure 2D–I show equivalent measurements; the only difference is that the former spectra were recorded using a narrowband VIS beam, whereas the latter employed a narrowband NIR beam. Since *H. halophila* PYP absorbs mainly in the 400–500 nm range, using a narrowband NIR upconversion beam ensures no direct optical or electronic excitation by the incident beams and avoids that the generated SFG signal itself falls in the absorption band of the chromophore. However, as shown in Figure 2D–I, the positions of the vibrational bands and the sign of Im­(χ_SPP_^(2)^) remain essentially unaffected by the choice of the upconversion wavelength within the uncertainty of the data retrieval procedure. Referring to Figure 2A, the characteristic out-of-plane and in-plane vibrational stretching modes of the chiral C^α^-H groups appeared both with and without the protein at 2890 and 2980 cm^–1^, respectively. At 2938 cm^–1^, a shoulder is visible for PYP, which we assigned to the Fermi resonance of methyl groups. Around 3270 cm^–1^, a broad chiral N–H stretching mode was observed both with and without the protein (Figure 2A), and additionally, the chiral amide I and amide II modes (Figure 2D,2G) were also detected. Based on the correlation proposed by Yan’s group, namely that a β-sheet-rich peptide/protein shows both chiral N–H stretching and chiral amide I modes,^16^ these results indicate that PLL itself already forms a well-ordered β-sheet at the surface. Circular dichroism (CD) analysis indicates that PLL in aqueous solution at pH7 adopts a mixed conformation dominated by antiparallel β-sheet (∼48%), with additional contributions from turns and disordered segments (Figure S3). Past experiments by others showed that at the fully charged states of PLL, i.e., at pH below 8, ∼60% of the polypeptide chain is characterized by β-sheet secondary structure and the remaining ∼40% of the chain stays unfolded. This ratio is related to the strong electrostatic repulsion between neighboring charge groups that forces elongation of the polypeptide rather than a fibril structure.^72,73^ VSFG measurements also reveal a predominantly well-ordered β-sheet structure, suggesting that assembly at the interface keeps the β-sheet organization. PYP adsorption on the surface of the β-pleated PLL sheet led to a red shift of the chiral N–H stretching mode (Figure 2A). However, the chiral amide I and II vibrational bands remained characteristic (Figure 2D,2G) in accordance with the dominant presence of β-strands in the huge PAS core (∼33% of the mass) and β-scaffold (∼33% of the mass) structural parts of the protein. The extracted chiral imaginary χ^2^ spectra for the corresponding PLL (Figure 2B purple line) and PYP-on-PLL (Figure 2C purple line) system showed a valley followed by a local peak at higher wavenumbers near 2960/2980 cm^–1^ and 3260/3310 cm^–1,^ hinting at neighboring vibrational bands with amplitudes of opposite sign. Since the C^α^-H group governs the chirality of the N–H moiety of the folded backbone,^74^ these doublets can be assigned to the ordered β-sheet secondary structure where the hydrogen bonds between N–H and C=O groups lie on the surface (XY plane in Figure 1). When the topmost layer is PLL (Figure 2E,2H), vibrational bands at 1563, 1603/1613, 1625, and 1678 cm^–1^ were obtained via MEM and spectral fitting methods (Table S1), which we assigned to COO^–^ asymmetric stretching (from PGA), NH_2_/NH_3_^+^ asymmetric deformation mode (from PLL), B_2_ and B_1_ mode of amide I oscillators, respectively. After the adsorption of PYP on the PLL surface (Figure 2F,2I), vibrational bands at 1557, 1566, 1592, 1614, 1629, and 1685 cm^–1^ emerged (Table S2). The resonance at 1557 cm^–1^ is most probably due to the chiral amide II mode. Ye et al. reported achiral and chiral SFG spectra of 39-residue peptide–membrane systems in the amide I/II region; however, no chiral amide II feature was observed.^75^ In contrast, a negative chiral amide II band near 1550 cm^–1^ was reported for pepsin at the air–water interface,^11^ which is particularly relevant here because pepsin also contains substantial β-sheet secondary structure. The bands at 1566 and 1592/1614 cm^–1^ correspond to COO^–^ asymmetric stretching and aromatic modes (ν­(C–C)), for which the aromatic CH stretching bands between 3000 and 3100 cm^–1^ are also visible (Figure 2A,2C, Table S2). Furthermore, we found positive amplitude for the B_1_ (ν_|_) and B_2_ (ν_⊥_) modes of the amide I oscillators at 1685 cm^–1^ and 1629 cm^–1^, respectively, and negative amplitude for the amide II band near 1557 cm^–1^ (Figure 2E,F). These chiral motives in the VSFG spectra are in accordance with the previously obtained chiral heterodyne-detected imaginary spectrum of concanavalin A and LK_7_β, which are also rich in β-sheet motifs.^14,76^ The feature observed near 1660 cm^–1^ may arise from an amide I mode associated with helical components. In the high-wavenumber region, the bands at 3115, 3211, 3282, and 3300 cm^–1^ are assigned to N–H stretching vibrations and related and/or coupled O–H stretching modes. Owing to the strong spectral overlap between peptide N–H and hydration-water O–H vibrations in this region, a clear separation of helical and β-sheet contributions is not possible based on these bands alone. However, the higher-wavenumber components likely reflect distinct hydrogen-bonding environments and may contain contributions from helical structures.^16^ The feature at 3387 cm^–1^ is assigned to the O–H stretching mode of chiral hydration water, consistent with an ordered hydration-shell water structure induced by the chiral protein assembly.^80^

**Figure 2:**
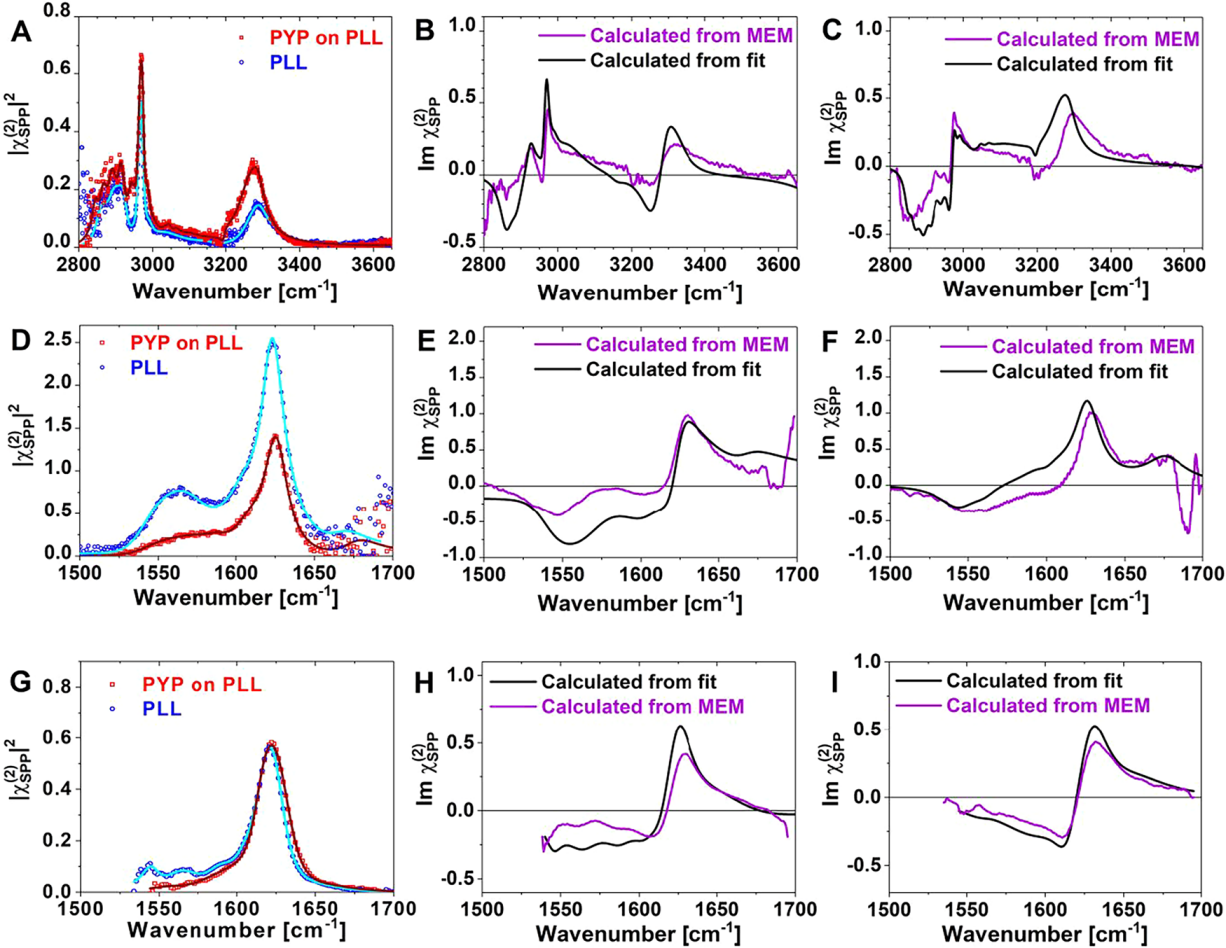
Chiral VSFG spectra of the PEI+(PGA+PLL)_6_ multilayer structure (“PLL”) and the PEI+(PGA+PLL)_6_+PYP multilayer structure (“PYP on PLL”), recorded in SPP polarization for the C–H/O-H region (A–C) and the amide I/II region (D–I). Narrowband upconversion wavelengths were 514 nm (A–F) and 1028 nm (G–I). Panels A, D, and G show normalized homodyne VSFG spectra with best-fit curves (eq 1). Panels B, E, and H show imaginary spectra for PLL, and panels C, F, and I correspond to PYP on PLL, with black lines indicating fits based on eq 1 and purple lines indicating MEM retrieval results.

### 3.2 Nano-FTIR Measurements

To obtain complementary spectral information with nanoscale spatial confinement, nano-FTIR measurements were carried out over the 800–2700 cm^–1^ spectral range at selected locations across the thin-film samples. Detailed analysis of the collected spectra in the range of 1250–1800 cm^–1^ was performed to reveal protein-related information. Figure 3A shows the averaged spectra of PLL and PYP on PLL samples recorded from a ∼ 25 nm spatial region. The spectra were obtained by plotting the second-harmonic optical phase shift after preprocessing. This analysis provides further evidence for interfacial β-sheet formation, as evidenced by the presence of characteristic vibrational bands at 1632 and 1688 cm^–1^ (Figure 3, blue line), similar to those observed by VSFG spectroscopy (see Figure 2D,2G) and CD measurements (Figure S3). The adsorption of PYP on the PLL surface induced substantial spectral changes (Figure 3, red line), exhibiting a strong characteristic amide I (1700–1600 cm^–1^) and amide II (1600–1500 cm^–1^) vibrational bands of PYP, and a strong symmetric stretching mode of COO̅ side chains around 1400 cm^–1^. These differences are more visible in the second derivative traces of Figure 3B, where we concentrate on the amide I and II regions. The so-obtained bands in this spectral region are primarily assigned to vibrational modes of the amino acid side chains and the characteristic secondary structures of PLL and PYP. Vibrational signatures of β-sheets were observed with a maximum at 1632 cm^–1^/1688 cm^–1^, and 1637 cm^–1^/1688 cm^–1^ for PLL and PYP on PLL, respectively. While the band corresponding to disordered structures appeared at 1658 cm^–1^ for PLL, the turns emerged at 1665 cm^–1^and 1670 cm^–1^ for PYP on PLL and PLL, respectively. Since PYP consists of approximately 45–50% β-sheet, 20–25% turns and loops, and only ∼20% helical content, the broad feature near 1660 cm^–1^ could plausibly arise from the helical elements of PYP. The presence of bands near 1560 and 1660 cm^–1^ further suggests that these helical segments are oriented at an angle relative to the substrate, allowing both amide I and amide II modes to be detected. Accordingly, the nano-FTIR results point to sensitivity to out-of-plane vibrational components and imply that the helical units are tilted with respect to the surface rather than being aligned strictly parallel or perpendicular to it. Importantly, the minima in the second derivative spectra of both PLL and PYP on PLL align closely with the frequencies which we assigned by VSFG spectroscopy (cf. Tables S1 and S2).

**Figure 3:**
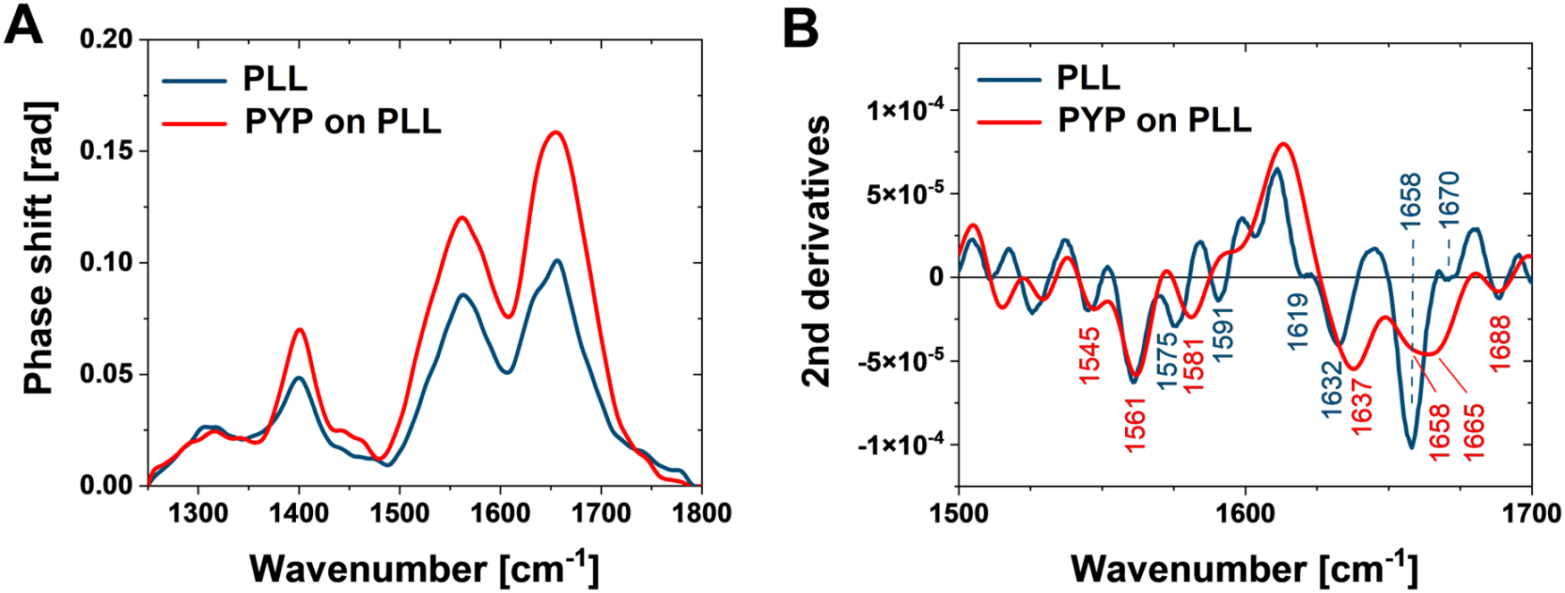
(A) Nano-FTIR spectra of PLL and PYP on PLL normalized by the spectrum of the Si substrate. (B) Second derivative of the near-field phase spectra in panel A (without any smoothing or additional preprocessing). The characteristic bands corresponding to PLL and PYP are marked.

### 3.3 Orientation of PYP Adsorbed on the PLL Surface

The VSFG signal of the PLL–PYP system depends on the orientation of the PYP molecule on the peptide surface. By comparing the theoretical spectra corresponding to different PYP orientations with the experimental spectra, we can determine the actual protein arrangement. In our study, we used the calculated amide I signal of the backbone amide groups of PYP for this purpose, similarly to what was proposed by Roeters et al.^20,64^ and developed further by Guo et al.^15,23^ A minor difference from their approach is that our model is based on averaging on a statistical sample (obtained from molecular dynamics simulations) and it takes into account, besides the intra- and interchain interactions of the amide groups, their interactions with protein side chains and the surrounding solvent molecules. Further differences from the above-mentioned approaches are that, instead of using two or more VSFG spectra with different polarization combinations, our method is based on the chiral SPP spectrum alone.

Nevertheless, the most significant difference stems from the fact that, in our case, not only the protein but also the peptide layers underneath are optically active, and neither the structure of these peptide layers nor the orientation of the protein on their surface is known. We used theoretical calculations to determine a suitable spatial arrangement of the peptides that yields an SPP intensity similar to the experimental one. This spectrum is then combined with that calculated for PYP with all the possible (θ,ψ) orientations

$$\chi_{\mathrm{SPP}}^{\mathrm{PLL}+\mathrm{PYP}}\left(\theta ,\psi \right)={\alpha \chi }_{\mathrm{SPP}}^{\mathrm{PLL}}+\left(1-\alpha \right)\chi_{\mathrm{SPP}}^{\mathrm{PYP}}\left(\theta ,\psi \right)$$

The actual α parameter is determined for each PYP orientation (θ,ψ) so that the RSS (residual sum of squares) error calculated from the experimental spectrum is as small as possible. The minima of the resulting RSS­(θ,ψ) function indicate the most probable surface orientation of the PYP.

Although this procedure seems conceptually straightforward, further approximations need to be implemented to overcome the existing difficulties. Fortunately, based on earlier measurements, two important approximations could be made that helped us build an effective model. The first is that each layer of the (PGA–PLL)_6_-PYP stack is very similar, with almost the same refractive indices with nm-scale thicknesses (i.e., molecular dimensions). Therefore, the peptide and protein layers can be considered as a single protein-like molecular film. With this in mind, a computational model was built to include not only the VSFG signal from the PYP layer, but also from the other underlying layers. The second approximation is based on the fact that the PGA layers do not contribute to the SPP signal due to their random structure,^38^ so we only need to include PYP and PLL in our model. In the case of the (PGA–PLL)_6_ system, direct structural information is not available; therefore, a model system must be created to calculate the SPP spectrum of PLL, whose spectrum closely approximates the measured one. As a preliminary step, an antiparallel β sheet consisting of six PLL chains is placed on a surface consisting of six disordered PGA chains. In selecting the PLL structure, secondary structural information obtained from experiments was utilized. A 200 ns molecular dynamics simulation is performed on the system, as outlined in the Methods section. Subsequently, we conducted SPP spectrum calculations using the two PLL chains positioned at the periphery of the β sheet, employing a data set comprising 200 structures derived from the latter half of the simulation. Given the absence of additional information regarding the structure of the PLL chains beyond their secondary structure, the calculated spectrum most analogous to the experiment in terms of RSS is selected through scanning the possible spatial orientations of these chains. This way, we obtain the approximate complex spectrum of the peptide layers.

Euler angles (φ, θ, ψ) were used to determine the relative orientation measured from the laboratory frame (**X**, **Y**, **Z**). For the reference orientation (φ = 0, θ = 0, ψ = 0), the axes of the molecular frame ((**x**, **y**, **z**) as defined in the experimental 3D structure of PYP (PDB ID: 2ZOI)) are parallel to those of the laboratory frame. Using surface symmetry, the signal was averaged for rotation around the **Z**-axis (φ), therefore, the two remaining Euler angles determine the relative orientation of the protein on the surface (θ,ψ). For these orientations, we calculated the RSS error of the theoretical and experimental curves as a measure of similarity, using an increment of 10 degrees for both angles. These values were plotted against the angles (θ,ψ) in Figure 4A.

**Figure 4:**
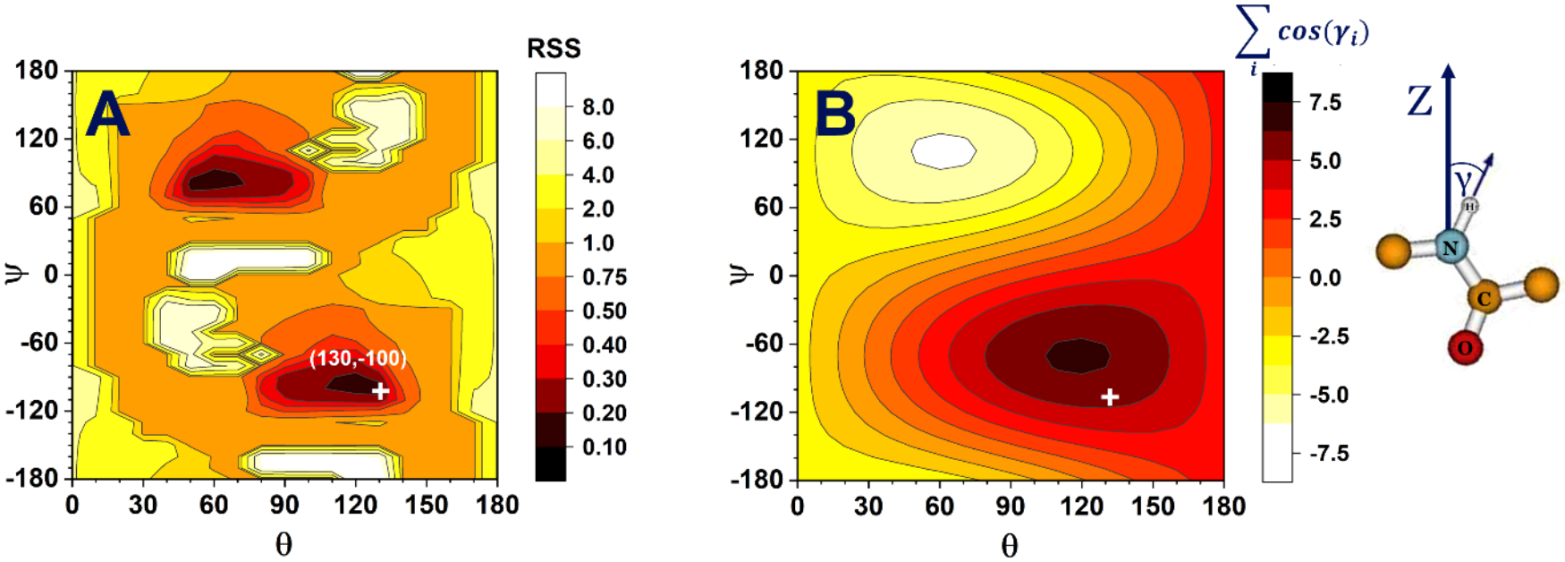
(A) RSS deviation of the experimental and theoretical SPP curves of the PLL+PYP system for the different (θ, ψ) orientations calculated in the amide I region. (B) Net orientation of the N–H bonds of PYP obtained from the simulation.

The two dark regions in Figure 4A show the orientations where the calculated spectra exhibit a high similarity to the experimental one. Up to this point, we used only one feature of the SPP spectrum, namely the intensity in the amide I region. However, for the true orientation, every geometric feature derived from the spectrum must match the features calculated from the MD simulation for a selected orientation. Therefore, we tried to find the proper geometric feature that would pick out the real orientation or orientations from the above-seen set of theoretical SPP intensities that is similar to the measured one in the amide I region. As derived from the MEM calculation of the imaginary part of the chiral VSFG signal, the dominant orientation of the N–H bonds from the backbone amide groups is the ‘upward’ orientation (positive Z direction, cf. Figure 2C). Assuming that the angle between the i-th N–H bond and the Z axis is γ_i_, we calculated the sum of the cos­(γ_i_)-s for the helical and β-strand regions of PYP (Figure 4B), incrementing the θ and ψ angles by 10 degrees. If this sum is positive, then the “upward” direction is dominant for the selected protein orientation. Comparing panels A and B of Figure 4, we can easily see that only one region fulfills this positivity criterion, namely the one at (θ = 130°, ψ = −100°). Fortunately, we obtained a single region for our protein, but for other systems, several regions may contribute to the spectra.

To verify the calculated orientation of PYP on the surface, we performed molecular dynamics simulations with a model system composed of a single PLL layer in an antiparallel β-sheet structure (in accordance with the experimental information obtained for the PLL structure) and a properly oriented PYP on its surface. As a first check, we determined the dipole vector of PYP in its initial (θ = 130°, ψ = −100°) orientation (Figure 5C), which is almost parallel to the positive Z direction, as expected for a protein on a positively charged surface. Using this initial position, we carried out a 200 ns-long NPT explicit water molecular dynamics simulation using Br- ions to compensate for the positive charges and NaBr at an additional concentration of 0.5 M. The Cα atoms of PLL were kept fixed during MD simulation, but the PYP molecule could move freely on the surface. We calculated the actual values of (θ,ψ) for 200 evenly spaced snapshots from the second half of the simulation (see Figure 5A), which remained close to the initial position, demonstrating the stability of this orientation on the surface.

**Figure 5:**
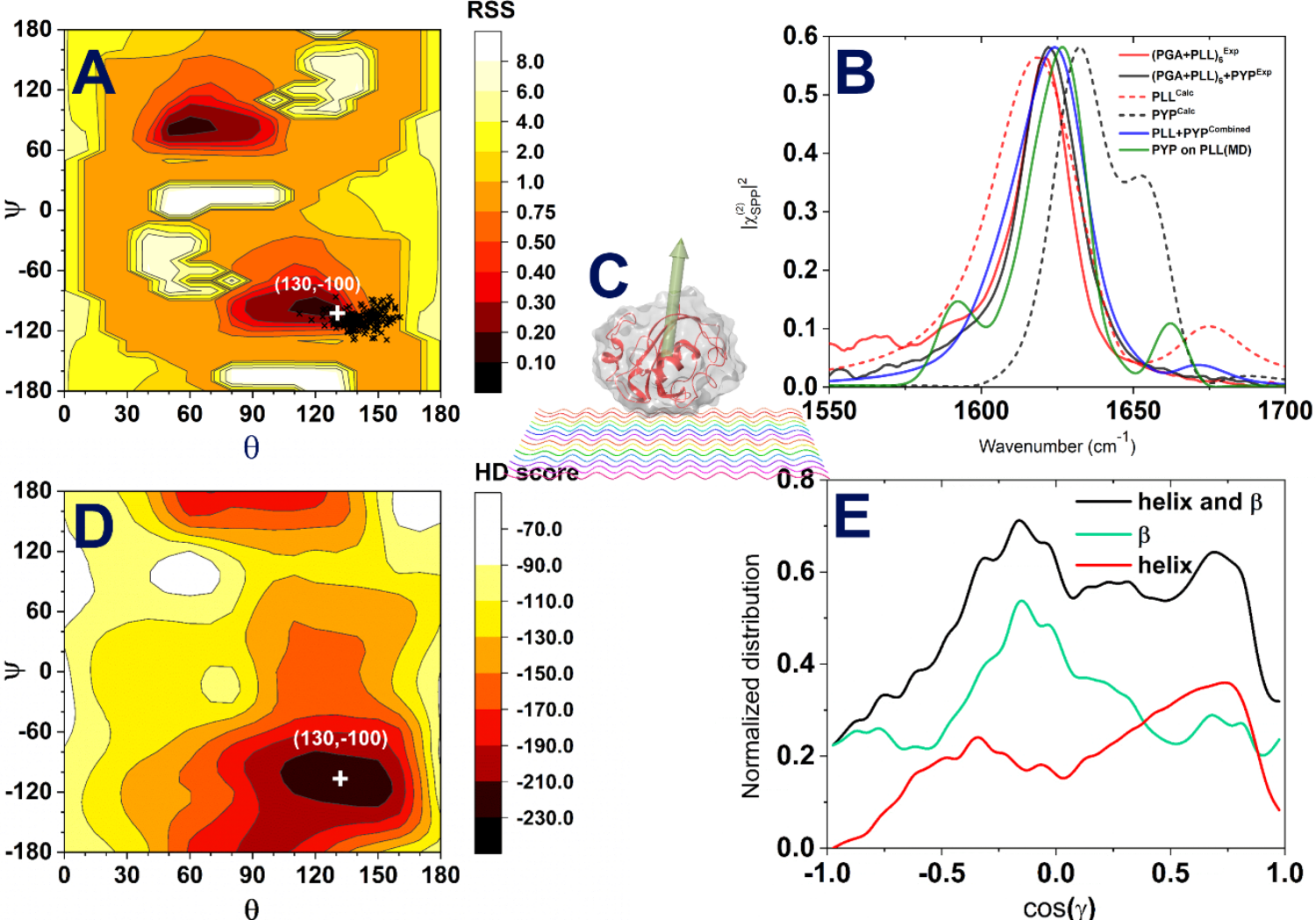
Results of the molecular dynamics simulation for the PYP–PLL and PLL systems. (A) Orientation of PYP on the PLL surface shown on the RMSD map from Figure 4A (+). (B) Comparison of the experimental (solid line) and theoretical (dashed line) SPP spectra for PYP on the PLL surface (black), the PLL surface (red), and the PYP on PLL spectrum obtained from PLL–PYP molecular dynamics simulation (green). The theoretical SPP spectrum of PYP was obtained for the orientation θ = 130°, ψ = −100°. (C) Dipole vector of PYP for the same orientation of the protein. (D) HADDOCK scores for the PYP–PLL complex obtained from the contact refinement protocol started with the PYP oriented in direction (θ, ψ). (E) Normalized distribution of the backbone N–H orientations (see Figure 4B for the definition of angle γ) for the helical and β-strand regions together (black) and their helical (red) and β-strand (green) components separately.

In addition, we also calculated the VSFG spectrum of PYP from the MD simulation of the PYP–PLL system. In this case, in addition to the intramolecular and PYP-water interactions, the PYP–PLL interactions were also taken into account in the calculation of the Hamiltonian in eq 3. Similarly to our first PLL–PYP calculation, these PYP and PLL signals were also superimposed. The spectrum obtained shows acceptable agreement with the experimental curve (see Figure 5B). Although the calculated curve (green) for the PYP–PLL system is somewhat blue-shifted and shows also differences in the 1650–1700 cm^–1^ region compared to the measured spectrum, leaving space for further parameter optimizations.

The dominant orientation of the backbone N–H bonds in the helical and β-strand regions remained also unchanged in the PYP–PLL simulation, as demonstrated in Figure 5E. The N to H vector of these bonds dominantly points to positive Z directions, as it is demonstrated by the distribution curves of cos­(γ) in Figure 5E. Separating contributions from the helical and the β-sheet regions, we can also see that the asymmetry stems dominantly from amide groups of the helices.

The contact of the PYP molecule with the PLL surface can also be considered as a protein–protein interaction and can be characterized using well-established computational tools of this field. In the present study, we used the HADDOCK program package, which is primarily designed for protein–protein docking but is also applicable for refining protein–protein contacts, which fits our present goal. We positioned the PYP molecule near the PLL surface used in our PLL–PYP MD simulation in all possible orientations using a 20° step grid in (θ, ψ) space. Each complex was refined using the protocol described in Section 2.3. The final HADDOCK scores are shown in Figure 5D. Although these scores do not directly represent binding energies, the more negative scores indicate more stable complexes. Importantly, the minimum of the HADDOCK score surface is very close to the value determined from the SPP VSFG spectrum (cf. Figure 5A).

Although the structural information from our model agrees well with the information derived from the chiral SPP VSFG measurement, we sought further experimental data to confirm that the interaction surface of PYP identified by us also occurs in other complexes and can be confirmed by other methods. Unfortunately, we could not find direct information on the complex-forming properties of PYP, thus, we collected indirect information from the crystal structure of PYP and the contact properties of an analogous PAS domain in a larger protein.

First, we checked PYP–PYP interactions, as an example of homodimerization typical for sensory proteins, in a PYP crystal that was identified by combined neutron diffraction crystallography (PDB ID: 2ZOI).^68^ Figure 6A presents a neighboring PYP–PYP pair from this crystal structure in which the upper PYP in red was aligned with the PYP in the PYP–PLL complex.

**Figure 6:**
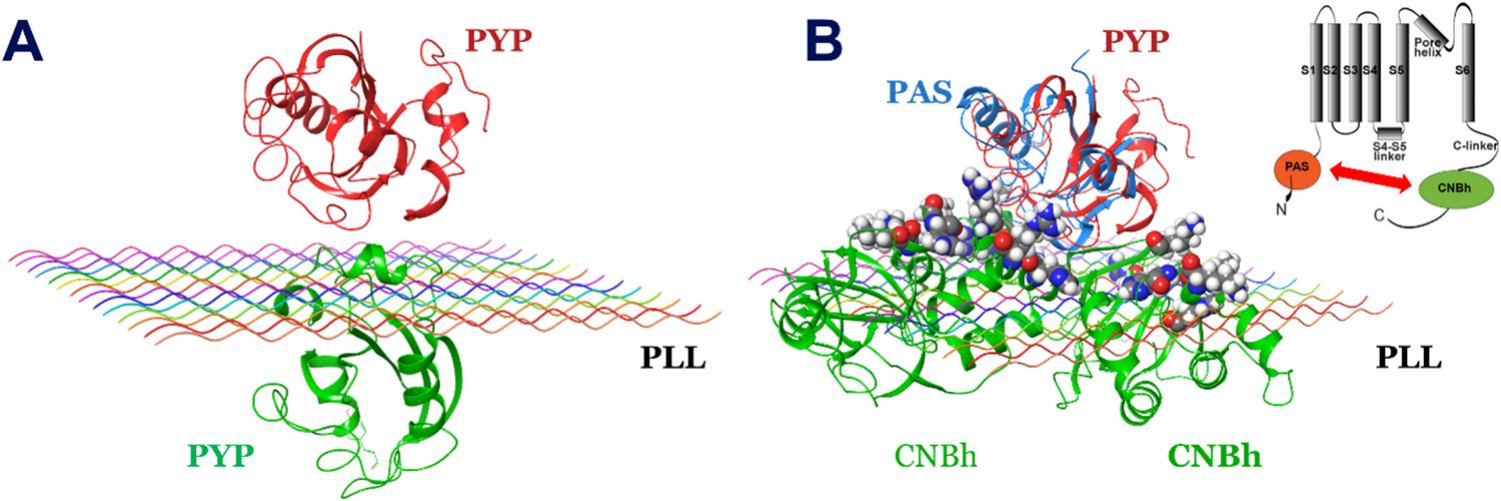
(A) Comparison of the PYP–PLL structure (PYP: red, PLL: tubes in rainbow coloring) with the relative orientation of a neighboring PYP–PYP pair in the crystal structure (PDB ID: 2ZOI). One of the PYP molecules (red) coincides with the PYP molecule from the PYP–PLL complex. (B) Comparison of the PYP–PLL structure with the X-ray structure of interacting PAS (blue) and CNBh (green) domains of the mouse KCNH channel (PDB ID: 4LLO).^77^ Atoms of the positively charged residues on the upper surface of CNBh domains close to PYP are shown in ball representation.

As can be seen, the interaction surface of PYP with PLL shows a clear overlap with one of the contact surfaces of PYP in the crystal structure. PYP belongs to the PAS domain family, often appearing in larger proteins. From these proteins, here we present a KCNH ion channel in which a PAS domain is located near the N-terminus, while the cyclic nucleotide-binding domain (CNBh) is positioned close to the C-terminus of the protein (see the inset in Figure 6B). The sequence of the PAS domain shows 45% similarity with PYP. The X-ray structure of the PAS-CNBh complex of the mouse KCNH ion channel is available in the Protein Data Bank (4LLO).^77^ Figure 6B shows two CNBh domains that interact with the PAS domain. One of them is the biological interaction partner of PAS (indicated by bolding its name), and the other belongs to a neighboring PAS-CNBh dimer in the crystal used in the X-ray crystallographic study. This structure is compared with the PYP–PLL complex by fitting the backbone of PYP and the PAS domain. This figure clearly demonstrates that the interaction surface of PYP in the PYP–PLL system closely resembles those of the PAS and CNBh domains.

### 3.4 Opposite Water Orientation at PLL and PYP Surfaces

Figure 7A shows the measured and fitted VSFG spectra of PLL with and without the adsorbed PYP layer on top in the spectral range of 2800 and 3700 cm^–1^ obtained in achiral, SSP polarization combination. The spectrum shows characteristic signatures of the methylene stretching bands of the Lys side chain, which shifted with PYP adsorption on the surface due to the nonpolar side chains of PYP. Additionally, the C^α^-H stretching band, which appears as a shoulder at 2980 cm^–1^, is also detected for both the polyelectrolyte and the protein top surface, but with a much higher vibrational amplitude in the case of the PLL–PYP system. As shown in Figure 7A for wavenumbers above 3000 cm^–1^, the interfacial water structure is very different when PYP is adsorbed on the PLL surface compared to the bare PLL interface.

**Figure 7:**
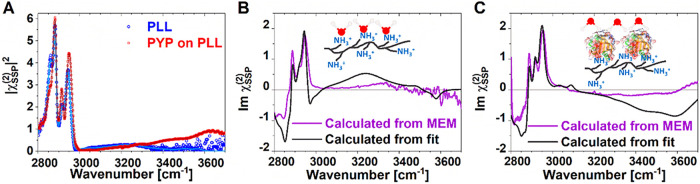
Achiral VSFG spectra of the PEI+(PGA+PLL)_6_ multilayer structure (“PLL”) and the PEI+(PGA+PLL)_6_+PYP multilayer structure (“PYP on PLL”), recorded in SSP polarization for the C–H/O-H region. Panel A shows normalized homodyne VSFG spectra with best-fit curves (eq 1). Panel B (C) shows imaginary spectra for PLL (PYP on PLL), with black lines indicating fits based on eq 1 and purple lines indicating MEM retrieval results. The insets show the molecular arrangements of the model systems.

The dominant water orientation at the PLL and the PLL–PYP surface can be determined using MEM decomposition of the corresponding experimental achiral SSP intensity spectrum. Alternatively, and to validate the MEM results, the imaginary part of χ ^2^ can also be calculated from the best-fit curve based on eq 1 (Figure 7B,7C). Above 3000 cm^–1^, a positive/negative sign of the broad O–H stretching band in the imaginary part of χ ^2^ represents a net hydrogen-up/down orientation.^78,79^ Thus, the bands at 3200 cm^–1^ and 3450 cm^–1^ for PLL suggest that water above the top of the polyelectrolyte exhibits a hydrogen-up orientation. Since PLL is positively charged and its side chains pointed upward in the experiments, such a hydrogen-up orientation at the PLL surface is expected, considering simple electrostatic effects. Upon PYP adsorption on the PLL surface, the O–H orientation flips to a hydrogen-down direction (cf. Figure 7C), most probably due to the side chains of PYP. Remarkably, a positive amplitude was also observed for the chiral O–H bending mode (Figure 2A–C), which in turn flipped to negative amplitude after PYP was adsorbed on the surface (Tables S1 and S2).

To cross-check the experimental observation of an opposite orientation of the water O–H orientation at the bare PLL interface compared to that at the PLL–PYP surface, we employed MD simulations. This provides an additional possibility to validate our model independently of our chiroptical results discussed above. For this purpose, we used finer sampling with 1000 snapshots from our PYP–PLL simulations to properly represent the water arrangements around the PLL and the PYP-covered PLL surface. Although the achiral VSFG signal of the water molecules does not originate exclusively from the first hydration shell (unlike the chiral water signal as it was revealed by Kontantinovsky et al.^80^), the distortion of the orientational distribution induced by the PLL or PYP surface is the strongest here, and therefore, we used this for comparison with the experimental results. The PLL surface is positively charged, which is compensated by Br^–^ ions in our case. The negative ions are often directly attached to the surface. Therefore, we considered the Br^–^ ions to be part of the surface, and their first hydration shell was added to the pool of water molecules that contact the PLL directly. We assume that the PLL surface was homogeneously covered by PYP with the same orientation as we described in Section 3.3. Nevertheless, due to packing effects, only the upper part of the protein (farther than 13 Å from the PLL surface in our case) could interact directly with water molecules. The orientation distribution of these water molecules (that is, the distribution of the cosine of the angle ξ between the dipole vector of the selected water molecules and the Z axis) is presented in Figure 8 for PLL and PYP, showing a well-defined, oppositely distorted shape that fits well with the experimental findings (Figure 7). Additionally, the distributions where the surface-associated Br^–^ ions are not taken into account are also presented. In the case of a PYP-covered surface, these negative ions have a minor influence. In contrast, when these ions at the PLL surface are to be ignored it gives rise to an even more distorted orientation distribution of the water molecules.

**Figure 8:**
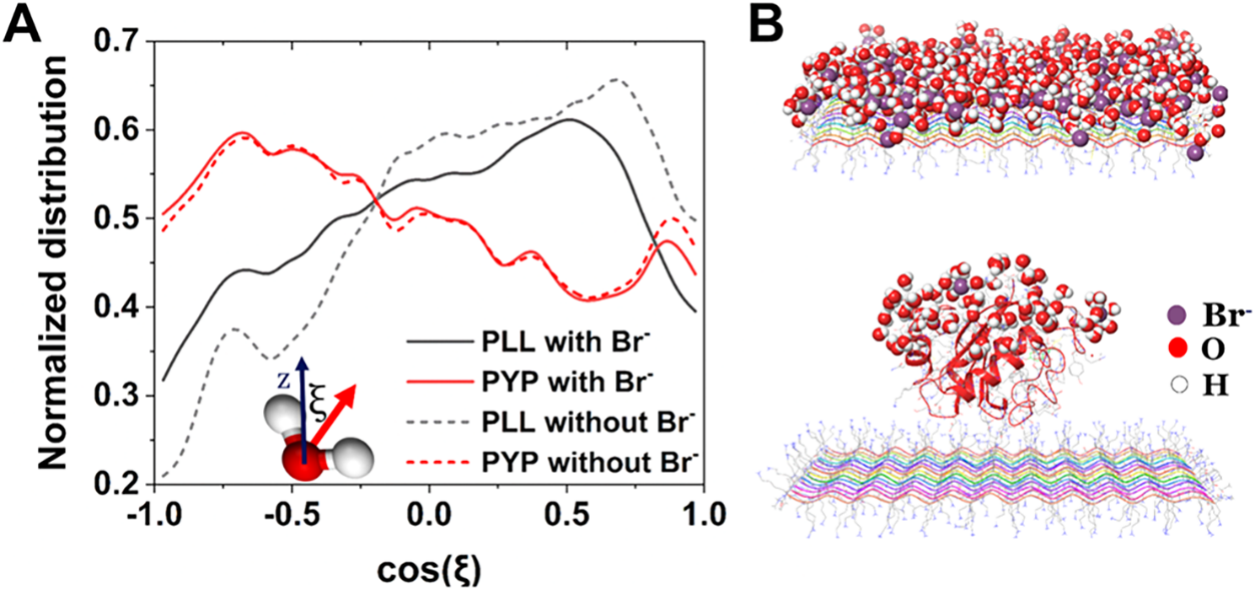
(A) Normalized distribution of the cosine of ξ angles (angle between the dipole vector of the selected water molecules and the Z axis). In this calculation, we used the water molecules in the first hydration shell of PLL and those belonging to the first solvation shell of the Br^–^ ions in close contact with PLL. (B) In the case of PYP, the water molecules were selected in the same way, but only those water molecules were taken into account that are farther than 13 Å from the PLL surface.

## 4 Conclusions

We studied in situ protein–protein interactions by combining nano-FTIR spectroscopy and chiral VSFG spectroscopy with MD simulations and calculations of VSFG spectra to assess macromolecular orientation at the interface. As a model for protein–protein interactions, we chose the adsorption of the photoactive yellow protein to a surface of poly-l-lysine. We found that both the bare PLL and the PYP-covered PLL surface exhibited ordered β-sheet secondary structure where the hydrogen bonds between N–H and C=O groups lie on the surface. The adsorption of PYP on the β-pleated PLL sheet led to a red shift of the N–H stretching mode. The root-mean-square deviation between measured and calculated chiral SPP VSFG spectra revealed two likely orientations of the PYP molecule on the PLL surface. The sign of the imaginary part of the measured χ^(2)^ was consistent with the calculated net orientation of the N–H bonds of PYP, and provided additional information that, in general, can help pick out the real orientation from the various possible alignments derived from the intensity spectrum alone. Our MD simulations confirmed that the extracted orientation of the adsorbed PYP molecule is consistent with stable docking on the PLL surface, and the changes in the calculated VSFG spectrum during molecular dynamics up to a duration of 200 ns remained small compared to the measured spectrum. Additionally, employing the HADDOCK protein–protein docking procedure for the PYP–PLL system also led to a very similar orientation. Furthermore, the interaction surface of PYP identified by us is consistent with the contact surfaces in (i) PYP–PYP interaction and orientation in homodimerization obtained for PYP crystals using neutron diffraction crystallography and (ii) the PAS-CNBh complex of the mouse KCNH ion channel, where the sequence of the PAS domain shows 45% similarity with PYP. Finally, our experiments revealed a flip of O–H orientation in the first hydration shell of PYP and the PLL surface compared to that of the bare PLL surface, which was confirmed by the net water dipole direction obtained from our MD simulations. Our combined experimental and theoretical strategy has high potential for determining the interfacial orientation of peptides and proteins and for identifying partner proteins in situ during protein–protein interactions.

## Supplementary Material



## Notes

Surface characterization of the multilayers using tapping mode AFM (Figure S1); AFM images and height profiles (Figure S2); circular dichroism (CD) spectrum of PLL (Figure S3); chiral vibrational mode assignments and the corresponding VSFG wavenumbers, spectral widths, and amplitudes of the multilayer films (Table S1 with PLL and Table S2 with PYP topmost layer); including the results of the nano-FTIR phase spectra; and (SI-MS-2026–03–31-final) (PDF)

## References

[1] Schreiber G. (2002). Kinetic Studies
of Protein–Protein Interactions. Curr.
Opin. Struct. Biol..

[2] Cheng F., Zhao J., Wang Y., Lu W., Liu Z., Zhou Y., Martin W. R., Wang R., Huang J., Hao T., Yue H., Ma J., Hou Y., Castrillon J. A., Fang J., Lathia J. D., Keri R. A., Lightstone F. C., Antman E. M., Rabadan R., Hill D. E., Eng C., Vidal M., Loscalzo J. (2021). Comprehensive Characterization of
Protein–Protein Interactions Perturbed by Disease Mutations. Nat. Genet..

[3] Lu H., Zhou Q., He J., Jiang Z., Peng C., Tong R., Shi J. (2020). Recent Advances in the Development
of Protein–Protein Interactions Modulators: Mechanisms and
Clinical Trials. Signal Transduct. Target Ther..

[4] Proteins at Interfaces II: Fundamentals and Applications. In ACS Symp. Ser.; Horbett, T. A. ; Brash, J. L. , Eds.; American Chemical Society: Washington, DC, 1995; Vol. 602.

[5] Laaser J. E., Skoff D. R., Ho J.-J., Joo Y., Serrano A. L., Steinkruger J. D., Gopalan P., Gellman S. H., Zanni M. T. (2014). Two-Dimensional
Sum-Frequency Generation Reveals Structure and Dynamics of a Surface-Bound
Peptide. J. Am. Chem. Soc..

[6] Centrone A. (2015). Infrared Imaging
and Spectroscopy Beyond the Diffraction Limit. Annu. Rev. Anal. Chem..

[7] Keilmann F., Hillenbrand R., Zayats A., Richard D. (2009). Near-Field Nanoscopy
by Elastic Light Scattering from a Tip. Nano
Opt. Field Opt. Microsc..

[8] Muller E. A., Pollard B., Bechtel H. A., Van Blerkom P., Raschke M. B. (2016). Infrared Vibrational Nanocrystallography
and Nanoimaging. Sci. Adv..

[9] Wang H.-F., Gan W., Lu R., Rao Y., Wu B.-H. (2005). Quantitative Spectral
and Orientational Analysis in Surface Sum Frequency Generation Vibrational
Spectroscopy (SFG-VS). Int. Rev. Phys. Chem..

[10] Hosseinpour S., Roeters S. J., Bonn M., Peukert W., Woutersen S., Weidner T. (2020). Structure and Dynamics
of Interfacial Peptides and
Proteins from Vibrational Sum-Frequency Generation Spectroscopy. Chem. Rev..

[11] Zhu X. D., Suhr H., Shen Y. R. (1987). Surface
Vibrational Spectroscopy
by Infrared-Visible Sum Frequency Generation. Phys. Rev. B.

[12] Fu L., Zhang Y., Wei Z., Wang H. (2014). Intrinsic Chirality
and Prochirality at Air/R-(+)- and S-(−)-Limonene Interfaces:
Spectral Signatures With Interference Chiral Sum-Frequency Generation
Vibrational Spectroscopy. Chirality.

[13] Okuno M., Ishibashi T. (2014). Chirality
Discriminated by Heterodyne-Detected Vibrational
Sum Frequency Generation. J. Phys. Chem. Lett..

[14] Okuno M., Ishibashi T. (2015). Heterodyne-Detected
Achiral and Chiral Vibrational
Sum Frequency Generation of Proteins at Air/Water Interface. J. Phys. Chem. C.

[15] Guo W., Lu T., Gandhi Z., Chen Z. (2021). Probing Orientations and Conformations
of Peptides and Proteins at Buried Interfaces. J. Phys. Chem. Lett..

[16] Yan E. C. Y., Fu L., Wang Z., Liu W. (2014). Biological Macromolecules
at Interfaces Probed by Chiral Vibrational Sum Frequency Generation
Spectroscopy. Chem. Rev..

[17] Chiang K.-Y., Matsumura F., Yu C.-C., Qi D., Nagata Y., Bonn M., Meister K. (2023). True Origin of Amide I Shifts Observed
in Protein Spectra Obtained with Sum Frequency Generation Spectroscopy. J. Phys. Chem. Lett..

[18] Boughton A. P., Yang P., Tesmer V. M., Ding B., Tesmer J. J. G., Chen Z. (2011). Heterotrimeric G Protein *β*
_1_
*γ*
_2_ Subunits Change Orientation
upon Complex Formation with G Protein-Coupled Receptor Kinase 2 (GRK2)
on a Model Membrane. Proc. Natl. Acad. Sci.
U.S.A..

[19] Hamm, P. ; Zanni, M. Concepts and Methods of 2D Infrared Spectroscopy, 1st ed.; Cambridge University Press, 2011.

[20] Roeters S. J., van Dijk C. N., Torres-Knoop A., Backus E. H. G., Campen R. K., Bonn M., Woutersen S. (2013). Determining
In Situ Protein Conformation
and Orientation from the Amide-I Sum-Frequency Generation Spectrum:
Theory and Experiment. J. Phys. Chem. A.

[21] Liang C., Louhivuori M., Marrink S. J., Jansen T. L. C., Knoester J. (2013). Vibrational
Spectra of a Mechanosensitive Channel. J. Phys.
Chem. Lett..

[22] Carr J. K., Wang L., Roy S., Skinner J. L. (2015). Theoretical Sum
Frequency Generation Spectroscopy of Peptides. J. Phys. Chem. B.

[23] Guo W., Zou X., Jiang H., Koebke K. J., Hoarau M., Crisci R., Lu T., Wei T., Marsh E. N. G., Chen Z. (2021). Molecular Structure
of the Surface-Immobilized Super Uranyl Binding Protein. J. Phys. Chem. B.

[24] Guo W., Lu T., Crisci R., Nagao S., Wei T., Chen Z. (2023). Determination
of Protein Conformation and Orientation at Buried Solid/Liquid Interfaces. Chem. Sci..

[25] Stuffle E. C., Johnson M. S., Watts K. J. (2021). PAS Domains
in Bacterial Signal Transduction. Curr. Opin.
Microbiol..

[26] Vreede J., Van Der Horst M. A., Hellingwerf K. J., Crielaard W., Van Aalten D. M. F. (2003). PAS Domains. J. Biol. Chem..

[27] Pellequer J.-L., Wager-Smith K. A., Kay S. A., Getzoff E. D. (1998). Photoactive Yellow
Protein: A Structural Prototype for the Three-Dimensional Fold of
the PAS Domain Superfamily. Proc. Natl. Acad.
Sci. U.S.A..

[28] Meyer T. E. (1985). Isolation
and Characterization of Soluble Cytochromes, Ferredoxins and Other
Chromophoric Proteins from the Halophilic Phototrophic Bacterium Ectothiorhodospira
Halophila. Biochim. Biophys. Acta, Bioenerg..

[29] Shima S., Sakai H. (1977). Polylysine Produced by Streptomyces. Agric.
Biol. Chem..

[30] Van
Beeumen J. J., Devreese B. V., Van Bun S. M., Hoff W. D., Hellingwerf K. J., Meyer T. E., Cusanovich M. A., Mcree D. E. (1993). Primary Structure of a Photoactive Yellow Protein from
the Phototrophic Bacterium *Ectothiorhodospira Halophila*, with Evidence for the Mass and the Binding Site of the Chromophore. Protein Sci..

[31] Baca M., Borgstahl G. E. O., Boissinot M., Burke P. M., Williams D. R., Slater K. A., Getzoff E. D. (1994). Complete Chemical Structure of Photoactive
Yellow Protein: Novel Thioester-Linked 4-Hydroxycinnamyl Chromophore
and Photocycle Chemistry. Biochemistry.

[32] Hennig S., Strauss H. M., Vanselow K., Yildiz Ö., Schulze S., Arens J., Kramer A., Wolf E. (2009). Structural and Functional
Analyses of PAS Domain Interactions of the Clock Proteins Drosophila
PERIOD and Mouse PERIOD2. PLoS Biol..

[33] Kim S., Nakasone Y., Takakado A., Yamazaki Y., Kamikubo H., Terazima M. (2021). A Unique Photochromic
UV-A Sensor Protein, Rc-PYP,
Interacting with the PYP-Binding Protein. Phys.
Chem. Chem. Phys..

[34] Khan J. S., Imamoto Y., Yamazaki Y., Kataoka M., Tokunaga F., Terazima M. (2005). A Biosensor in the
Time Domain Based on the Diffusion
Coefficient Measurement: Intermolecular Interaction of an Intermediate
of Photoactive Yellow Protein. Anal. Chem..

[35] Park T., Jeong J., Kim S. (2006). Current Status
of Polymeric Gene
Delivery Systems☆. Adv. Drug Delivery
Rev..

[36] Mazia D., Schatten G., Sale W. (1975). Adhesion of Cells to
Surfaces Coated
with Polylysine. Applications to Electron Microscopy. J. Cell Biol..

[37] Porcel C. H., Izquierdo A., Ball V., Decher G., Voegel J.-C., Schaaf P. (2005). Ultrathin
Coatings and (Poly­(Glutamic Acid)/Polyallylamine)
Films Deposited by Continuous and Simultaneous Spraying. Langmuir.

[38] Krekic S., Mero M., Kuhl M., Balasubramanian K., Dér A., Heiner Z. (2023). Photoactive Yellow Protein Adsorption
at Hydrated Polyethyleneimine and Poly-l-Glutamic Acid Interfaces. Molecules.

[39] Panayotov I. V., Collart-Dutilleul P.-Y., Salehi H., Martin M., Végh A., Yachouh J., Vladimirov B., Sipos P., Szalontai B., Gergely C., Cuisinier F. J. G. (2014). Sprayed
Cells and Polyelectrolyte
Films for Biomaterial Functionalization: The Influence of Physical
PLL-PGA Film Treatments on Dental Pulp Cell Behavior: Sprayed Cells
and Polyelectrolyte Films for Biomaterial Functionalization. Macromol. Biosci..

[40] Picart C., Ladam G., Senger B., Voegel J.-C., Schaaf P., Cuisinier F. J. G., Gergely C. (2001). Determination of Structural Parameters
Characterizing Thin Films by Optical Methods: A Comparison between
Scanning Angle Reflectometry and Optical Waveguide Lightmode Spectroscopy. J. Chem. Phys..

[41] Michel M., Toniazzo V., Ruch D., Ball V. (2012). Deposition Mechanisms
in Layer-by-Layer or Step-by-Step Deposition Methods: From Elastic
and Impermeable Films to Soft Membranes with Ion Exchange Properties. ISRN Mater. Sci..

[42] Robledo I. P., Maciel-Escudero C., Schnell M., Mester L., Aizpurua J., Hillenbrand R. (2025). Theoretical Description of Infrared Near-Field Spectroscopy
of In- and Out-of-Plane Molecular Vibrations in Thin Layers. ACS Photonics.

[43] Schade U., Röseler A., Korte E. H., Bartl F., Hofmann K. P., Noll T., Peatman W. B. (2002). New Infrared Spectroscopic Beamline
at BESSY II. Rev. Sci. Instrum..

[44] Veber A., Puskar L., Kneipp J., Schade U. (2024). Infrared Spectroscopy
across Scales in Length and Time at BESSY II. J. Synchrotron Radiat..

[45] Nečas D., Klapetek P. (2012). Gwyddion: An Open-Source
Software for SPM Data Analysis. Open Phys..

[46] Heiner Z., Petrov V., Mero M. (2017). Compact, High-Repetition-Rate Source
for Broadband Sum-Frequency Generation Spectroscopy. APL Photonics.

[47] Heiner Z., Wang L., Petrov V., Mero M. (2019). Broadband Vibrational
Sum-Frequency Generation Spectrometer at 100 kHz in the 950–1750
Cm ^–1^ Spectral Range Utilizing a LiGaS _2_ Optical Parametric Amplifier. Opt. Express.

[48] Heiner Z., Der A., Petrov V., Mero M. (2024). Nonlinear Vibrational Spectrometer
for Bioapplications Featuring Narrowband 1-Μm Pulses and a Recycled
OPA Pump Beam. Opt. Express.

[49] Yesudas F., Mero M., Kneipp J., Heiner Z. (2018). Vibrational Sum-Frequency
Generation Spectroscopy of Lipid Bilayers at Repetition Rates up to
100 kHz. J. Chem. Phys..

[50] Sovago M., Vartiainen E., Bonn M. (2009). Determining Absolute Molecular Orientation
at Interfaces: A Phase Retrieval Approach for Sum Frequency Generation
Spectroscopy. J. Phys. Chem. C.

[51] De
Beer A. G. F., Samson J.-S., Hua W., Huang Z., Chen X., Allen H. C., Roke S. (2011). Direct Comparison of
Phase-Sensitive Vibrational Sum Frequency Generation with Maximum
Entropy Method: Case Study of Water. J. Chem.
Phys..

[52] Moloney E. G., Azam M. S., Cai C., Hore D. K. (2022). Vibrational Sum
Frequency Spectroscopy of Thin Film Interfaces. Biointerphases.

[53] Hamm P., Lim M., Hochstrasser R. M. (1998). Structure of the Amide I Band of
Peptides Measured by Femtosecond Nonlinear-Infrared Spectroscopy. J. Phys. Chem. B.

[54] Gorbunov R. D., Kosov D. S., Stock G. (2005). *Ab
Initio* -Based
Exciton Model of Amide I Vibrations in Peptides: Definition, Conformational
Dependence, and Transferability. J. Chem. Phys..

[55] Ham S., Cho M. (2003). Amide I Modes in the *N* -Methylacetamide Dimer and
Glycine Dipeptide Analog: Diagonal Force Constants. J. Chem. Phys..

[56] Meister K., Roeters S. J., Paananen A., Woutersen S., Versluis J., Szilvay G. R., Bakker H. J. (2017). Observation
of pH-Induced
Protein Reorientation at the Water Surface. J. Phys. Chem. Lett..

[57] Okuno M., Ishibashi T. (2018). Bulk-or-Interface
Assignment of Heterodyne-Detected
Chiral Vibrational Sum Frequency Generation Signal by Its Polarization
Dependence. J. Chem. Phys..

[58] Lu C., Wu C., Ghoreishi D., Chen W., Wang L., Damm W., Ross G. A., Dahlgren M. K., Russell E., Von Bargen C. D., Abel R., Friesner R. A., Harder E. D. (2021). OPLS4: Improving
Force Field Accuracy on Challenging Regimes of Chemical Space. J. Chem. Theory Comput..

[59] Schrödinger Release 2021–4 . Desmond Molecular Dynamics System, D. E. Shaw Research, New York NY, 2021 Maestro-Desmond Interoperability Tools; Schrödinger: New York, NY, 2021.

[60] Bowers, K. J. ; Sacerdoti, F. D. ; Salmon, J. K. ; Shan, Y. ; Shaw, D. E. ; Chow, E. ; Xu, H. ; Dror, R. O. ; Eastwood, M. P. ; Gregersen, B. A. ; Klepeis, J. L. ; Kolossvary, I. ; Moraes, M. A. Molecular Dynamics---Scalable Algorithms for Molecular Dynamics Simulations on Commodity Clusters; Proceedings of the 2006 ACM/IEEE conference on Supercomputing - SC ’06, ACM Press: Tampa, FL, 2006 84.

[61] Martyna G. J., Tobias D. J., Klein M. L. (1994). Constant
Pressure Molecular Dynamics
Algorithms. J. Chem. Phys..

[62] Tuckerman M., Berne B. J., Martyna G. J. (1992). Reversible
Multiple Time Scale Molecular
Dynamics. J. Chem. Phys..

[63] Essmann U., Perera L., Berkowitz M. L., Darden T., Lee H., Pedersen L. G. (1995). A Smooth Particle
Mesh Ewald Method. J. Chem. Phys..

[64] Roeters S.
J., Strunge K., Pedersen K. B., Golbek T. W., Bregnhøj M., Zhang Y., Wang Y., Dong M., Nielsen J., Otzen D. E., Schiøtt B., Weidner T. (2023). Elevated Concentrations
Cause Upright Alpha-Synuclein Conformation at Lipid Interfaces. Nat. Commun..

[65] Marcus Schwarting. VSFG-Bellerephon. https://github.com/meschw04/vsfg-bellerephon (accessed December 6, 2023).

[66] Michaud-Agrawal N., Denning E. J., Woolf T. B., Beckstein O. (2011). MDAnalysis:
A Toolkit for the Analysis of Molecular Dynamics Simulations. J. Comput. Chem..

[67] Gowers, R. ; Linke, M. ; Barnoud, J. ; Reddy, T. ; Melo, M. ; Seyler, S. ; Domański, J. ; Dotson, D. ; Buchoux, S. ; Kenney, I. ; Beckstein, O. MDAnalysis: A Python Package for the Rapid Analysis of Molecular Dynamics Simulations. 2016, pp 98–105 10.25080/Majora-629e541a-00e.

[68] Yamaguchi S., Kamikubo H., Kurihara K., Kuroki R., Niimura N., Shimizu N., Yamazaki Y., Kataoka M. (2009). Low-Barrier Hydrogen
Bond in Photoactive Yellow Protein. Proc. Natl.
Acad. Sci. U.S.A..

[69] Honorato R. V., Koukos P. I., Jiménez-García B., Tsaregorodtsev A., Verlato M., Giachetti A., Rosato A., Bonvin A. M. J. J. (2021). Structural Biology in the Clouds:
The WeNMR-EOSC Ecosystem. Front. Mol. Biosci..

[70] Dominguez C., Boelens R., Bonvin A. M. J. J. (2003). HADDOCK:
A Protein–Protein
Docking Approach Based on Biochemical or Biophysical Information. J. Am. Chem. Soc..

[71] Van
Zundert G. C. P., Rodrigues J. P. G. L.
M., Trellet M., Schmitz C., Kastritis P. L., Karaca E., Melquiond A. S. J., Van Dijk M., De Vries S. J., Bonvin A. M. J. J. (2016). The HADDOCK2.2
Web Server: User-Friendly Integrative Modeling of Biomolecular Complexes. J. Mol. Biol..

[72] Batys P., Morga M., Bonarek P., Sammalkorpi M. (2020). pH-Induced
Changes in Polypeptide Conformation: Force-Field Comparison with Experimental
Validation. J. Phys. Chem. B.

[73] Mikhonin A. V., Myshakina N. S., Bykov S. V., Asher S. A. (2005). UV Resonance Raman
Determination of Polyproline II, Extended 2.5 _1_ -Helix,
and β-Sheet Ψ Angle Energy Landscape in Poly- l -Lysine
and Poly- l -Glutamic Acid. J. Am. Chem. Soc..

[74] Hu X.-H., Fu L., Hou J., Zhang Y.-N., Zhang Z., Wang H.-F. (2020). N–H
Chirality in Folded Peptide LK _7_ β Is Governed by
the C _α_ – H Chirality. J. Phys. Chem. Lett..

[75] Tan J., Zhang J., Luo Y., Ye S. (2019). Misfolding of a Human
Islet Amyloid Polypeptide at the Lipid Membrane Populates through
β-Sheet Conformers without Involving α-Helical Intermediates. J. Am. Chem. Soc..

[76] Perets E. A., Konstantinovsky D., Fu L., Chen J., Wang H.-F., Hammes-Schiffer S., Yan E. C. Y. (2020). Mirror-Image Antiparallel β-Sheets
Organize Water Molecules into Superstructures of Opposite Chirality. Proc. Natl. Acad. Sci. U. S.A..

[77] Morais-Cabral J. H., Robertson G. A. (2015). The Enigmatic
Cytoplasmic Regions of KCNH Channels. J. Mol.
Biol..

[78] Nojima Y., Suzuki Y., Yamaguchi S. (2017). Weakly Hydrogen-Bonded
Water Inside
Charged Lipid Monolayer Observed with Heterodyne-Detected Vibrational
Sum Frequency Generation Spectroscopy. J. Phys.
Chem. C.

[79] Nihonyanagi S., Mondal J. A., Yamaguchi S., Tahara T. (2013). Structure and Dynamics
of Interfacial Water Studied by Heterodyne-Detected Vibrational Sum-Frequency
Generation. Annu. Rev. Phys. Chem..

[80] Konstantinovsky D., Perets E. A., Santiago T., Velarde L., Hammes-Schiffer S., Yan E. C. Y. (2022). Detecting the
First Hydration Shell Structure around
Biomolecules at Interfaces. ACS Cent. Sci..

