# Supplementary material for: PAS-Domain Protein Orientation at a Polyelectrolyte Surface Revealed by Infrared Nanospectroscopy, Chiral Vibrational Spectroscopy, and Molecular Dynamics Simulations
Source: Biomacromolecules. 2026 Apr 30;27(6):3569–81. doi: 10.1021/acs.biomac.5c02743 (PMC13250908; doi:10.1021/acs.biomac.5c02743)
Supplement: Supplementary file 1 [file bm5c02743_si_001.pdf]

Supplementary Materials

PAS-domain protein orientation at a polyelectrolyte surface revealed by infrared nanospectroscopy, chiral vibrational spectroscopy, and molecular dynamics simulations

*Ferenc Bogár,[1, *] Montserrat Román Quintero,[2,3] János Horváth,[4,5] Zoltán Násztor,[4] Mark Mero,[6] Szilvia Krekic,[4] Alexander Veber,[3,7] Ljiljana Puskar,[7] András Dér,[4] and Zsuzsanna Heiner[2,3, *]*

[1] HUN-REN-SZTE Biomimetic Systems Research Group, Department of Medical Chemistry, University of Szeged, H-6720 Szeged, Hungary

[2] School of Analytical Sciences Adlershof, Humboldt-Universität zu Berlin, 12489 Berlin, Germany

[3] Department of Chemistry, Humboldt-Universität zu Berlin, 12489 Berlin, Germany

[4] HUN-REN Biological Research Centre, Institute of Biophysics, H-6726 Szeged, Hungary

[5] Doctoral School of Physics, University of Szeged, H-6720 Szeged, Hungary

[6] Max Born Institute for Nonlinear Optics and Short Pulse Spectroscopy, 12489 Berlin, Germany

[7] Institute for Electronic Structure Dynamics, Helmholtz-Zentrum Berlin für Materialien und Energie GmbH, 12489 Berlin, Germany

[*] Corresponding authors: bogar@sol.cc.u-szeged.hu, zsuzsanna.heiner@hu-berlin.de

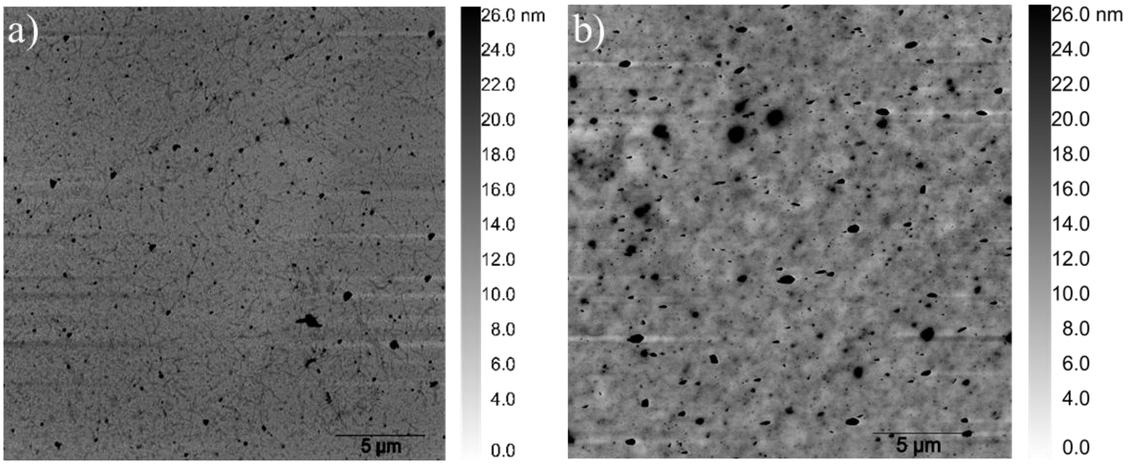

Figure S1 *Surface characterization of a) PEI+(PGA+PLL)$_6$ and b) PEI+(PGA+PLL)$_6$+PYP multilayers using tapping mode AFM. a) Before PYP was deposited, we observed a surface topography with fibers. b) After coating the surface with the PYP protein, a coarse surface is observable. The suggested fibers observed in a) are no longer visible; therefore, it can be said that a molecular layer covers the surface. Furthermore, the roughness after PYP coverage appears slightly higher; however, because the height range is similar to that before PYP deposition, it can be concluded that the protein indeed forms a homogeneous layer on the topmost layer of PLL on the Si surface.*

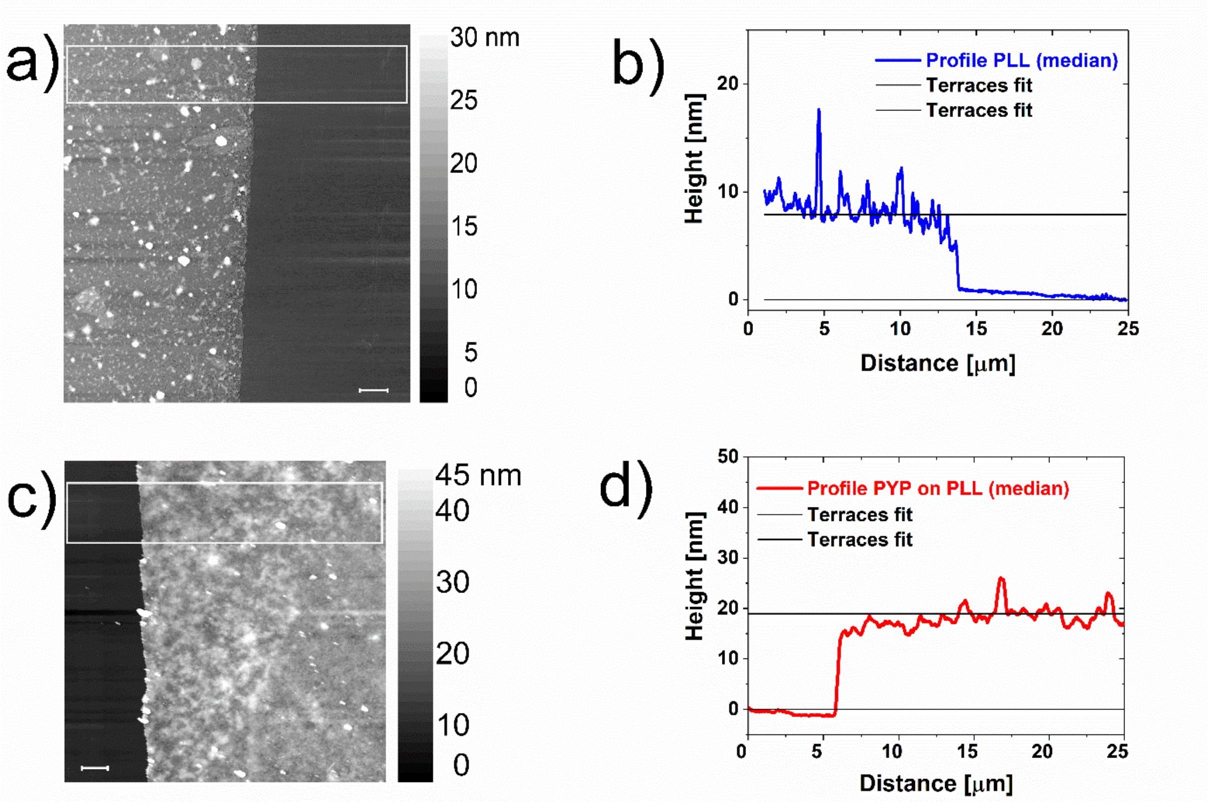

Figure S2 *AFM images and height profiles across the a,b) PEI+(PGA+PLL)$_6$ and the c,d) PEI+(PGA+PLL)$_6$+PYP film-substrate boundary, showing a total film thickness of 7.9 ± 0.1 nm and 18.9 ± 0.2 nm, respectively. Scale bars: 2 µm.*

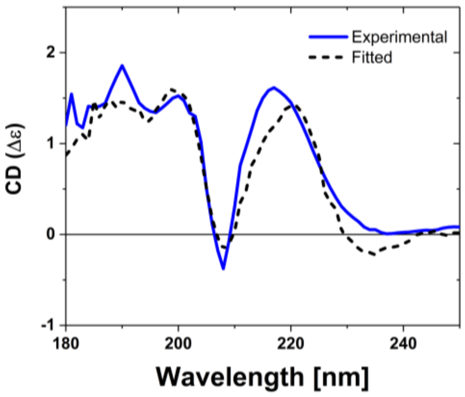

Figure S3 *Circular dichroism (CD) spectrum of PLL acquired in the bulk phase at pH 7 using an Olis-DSM20 spectrophotometer. The measurement was performed using a rectangular cell with an optical path of 1mm and at a concentration of 1mg/mL in H_2O at 22 °C. The graph indicates that PLL predominantly adopts 48% antiparallel β-sheet, 15.3% turn, and 36.7% other conformations, as stated by the web server for secondary structure determination and fold recognition from circular dichroism spectra, BeStSel– ELTE Eötvös Loránd University, Budapest, Hungary.*

Table S1 *Chiral vibrational mode assignments and the corresponding VSFG wavenumbers, spectral widths, and amplitudes of the PEI+(PGA+PLL)$_6$ multilayer film (with PLL as the topmost layer), (A) in the spectral region between 1540 and 1700 cm^{-1}, and (B) between 2800 and 3600 cm^{-1}. The rightmost column lists vibrational band wavenumbers obtained from the second derivative of the nano-FTIR phase spectra.*

**Band assignments, chiral (SPP) PLL pH 7**	**ω_v [cm^{-1}]**	**Γ_v**	**Q_v**	**ω_{IR} [cm^{-1}]**
**A) spectral region between 1540 and 1700 cm^{-1}**				
Amide II	1545	6.9	-1.3	1545
Amide II	1563	14.9	-3.4	1561
ν_{AS} COO^{-} *	1587	13.1	-2.3	1575
δ NH_2	1603	9.5	-1.3	1591
δ_{as} NH_3^+	1613	9.7	-2.9	1519
Amide I, B2 mode of antiparallel β-sheet	1625	10.8	7.7	1632
O-H bending	1648	30.9	3.1	
Amide I, disordered structure				1658
Amide I, turns				1670
Amide I, B1 mode of antiparallel β-sheet	1676	11.2	0.2	1688
**B) spectral region between 2800 and 3600 cm^{-1}**				
ν_S CH_2, Lys side chain	2860	22.0	-7.6	
ν_{Fermi} CH_2, Lys side chain	2889	20.0	-3.8	
ν_{AS} CH_2, Lys side chain	2928	19.9	5.6	
ν C^{α}-H, backbone	2963	12.7	-9.2	
ν C^{α}-H, backbone	2970	11.8	10.4	
ν C^{α}-H mixed with ν CH_2 modes	2995	13.3	1.4	
NH / NH_3^+ of Lys side-chain	3026	60.1	8.0	
amide B / strongly H-bonded N–H	3100	139.4	22	
N–H / hydration-water O–H	3254	50.3	-18.1	
N–H / hydration-water O–H	3309	51.4	16.6	
chiral hydration water ν O–H	3407	99.7	10.3	

Table S2 *Chiral vibrational mode assignments and corresponding VSFG wavenumbers, spectral widths, and amplitudes of the PEI+(PGA+PLL)$_6$+PYP multilayer film (with PYP as the topmost layer), (A) in the spectral region between 1540 and 1700 cm^{-1}, and (B) between 2800 and 3600 cm^{-1}.The rightmost column lists vibrational band wavenumbers obtained from the second derivative of the nano-FTIR phase spectra.*

**Band assignments, chiral (SPP) PLL+PYP pH 7**	**ω_v [cm^{-1}]**	**Γ_v**	**Q_v**	**ω_{IR}[cm^{-1}]**
**A) spectral region between 1540 and 1700 cm^{-1}**				
Amide II				1545
Amide II	1559	18.5	-1.2	1561
ν_{AS} COO^{-*}(Glu, Asp)	1578	50.1	-1.2	1581
δ NH_2 / νC-C (Tyr, Trp)	1597	29.0	-8.0	1599
δ_{as} NH_3^+ / νC-C (Tyr, Trp)	1614	11.0	-4.6	
Amide I, B2 mode of antiparallel β-sheet	1627	13.8	11.3	1537
O-H bending	1640	29.0	0.8	
Amide I, α-helix	1660	39.2	3.0	1658/1665
Amide I, B1 mode of antiparallel β-sheet	1679	10.9	0.2	1688
**B) spectral region between 2800 and 3600 cm^{-1}**				
ν_S CH_2	2852	22.9	-7.5	
ν_S CH_3	2887	25.5	-9.7	
ν_{AS} CH_2	2910	8.1	-1.1	
ν_{Fermi} CH_3	2938	8.0	-0.8	
ν_{AS} CH_3	2965	11.9	-5.2	
ν C^{α}-H	2973	8.0	4.1	
ν C^{α}-H mixed with ν CH_2 modes	2989	13.1	2.8	
aromatic ν C-H (Tyr, Phe, and Trp)	3009	18.6	1.8	
aromatic ν C-H (Tyr, Phe, and Trp)	3047	10.4	0.7	
aromatic ν C-H (Tyr, Phe, and Trp)	3068	8.0	0.2	
amide B / strongly H-bonded N–H	3115	88.7	17.8	
N–H / hydration-water O–H	3211	43.4	-17.3	
N–H / hydration-water O–H	3282	15.4	2.0	
N–H / hydration-water O–H	3300	41.7	14.9	
chiral hydration water ν O–H	3387	119.9	13.4	
